# Defective T Memory Cell Differentiation after Varicella Zoster Vaccination in Older Individuals

**DOI:** 10.1371/journal.ppat.1005892

**Published:** 2016-10-20

**Authors:** Qian Qi, Mary M. Cavanagh, Sabine Le Saux, Lisa E. Wagar, Sally Mackey, Jinyu Hu, Holden Maecker, Gary E. Swan, Mark M. Davis, Cornelia L. Dekker, Lu Tian, Cornelia M. Weyand, Jörg J. Goronzy

**Affiliations:** 1 Department of Medicine, Division of Immunology and Rheumatology, Stanford University, Stanford, California, United States of America; 2 Department of Medicine, VAPAHCS, Palo Alto, California, United States of America; 3 Institute for Immunity, Transplantation, and Infection, Stanford University, Stanford, California, United States of America; 4 Department of Pediatrics-Infectious Diseases, Stanford University, Stanford, California, United States of America; 5 Department of Medicine, Stanford Prevention Research Center, Stanford University, Stanford, California, United States of America; 6 Department of Biomedical Data Science, Stanford University, Stanford, California, United States of America; Baylor College of Medicine, UNITED STATES

## Abstract

Vaccination with attenuated live varicella zoster virus (VZV) can prevent zoster reactivation, but protection is incomplete especially in an older population. To decipher the molecular mechanisms underlying variable vaccine responses, T- and B-cell responses to VZV vaccination were examined in individuals of different ages including identical twin pairs. Contrary to the induction of VZV-specific antibodies, antigen-specific T cell responses were significantly influenced by inherited factors. Diminished generation of long-lived memory T cells in older individuals was mainly caused by increased T cell loss after the peak response while the expansion of antigen-specific T cells was not affected by age. Gene expression in activated CD4 T cells at the time of the peak response identified gene modules related to cell cycle regulation and DNA repair that correlated with the contraction phase of the T cell response and consequently the generation of long-lived memory cells. These data identify cell cycle regulatory mechanisms as targets to reduce T cell attrition in a vaccine response and to improve the generation of antigen-specific T cell memory, in particular in an older population.

## Author Summary

Vaccination is one of the most successful medical interventions, but it loses its effectiveness in an older population that is of particular risk for infectious diseases. Shingles, caused by the reactivation of the chickenpox virus, is a prime example. Nearly every second individual has experienced shingles by the age of 80 years, and the shingles vaccine is only partially protective. Attempts to improve the vaccine response are mostly empiric. Vaccinations induce a rapid expansion of antigen-specific T cells with frequencies peaking after one to two weeks. Most expanded T cells die after the peak response, and only few T cells survive to provide protection from infection or, as in case of shingles, from reactivation of latent viruses. Most vaccine studies have focused on the early stages of the response; how T cells are activated and expand. Surprisingly, in our study with the shingle vaccine, T cell survival after the peak response was the major factor determining memory T cell frequencies. T cell attrition was increased with age, independent of genetic predisposition. Using systems biology tools we found several pathways involved in T cell division and DNA repair that could be targeted to improve T cell survival and thereby increase the effectiveness of vaccination.

## Introduction

Herpes zoster, caused by the reactivation of the varicella zoster virus (VZV), affects one in two to three adults over the course of life. By far the biggest risk factor for VZV reactivation is age with the annual incidence increasing from 0.3% in adults at the age of 50 to more than 2% in adults over the age of 75 years [1,2]. In 2006, the FDA licensed a vaccine to protect against VZV reactivation, Zostavax. Several studies have described the vaccine in epidemiological terms of protection, safety, and efficacy [3–5]; in clinical trials, the efficacy of the vaccination to prevent VZV reactivation declines from 70% for 50–59 year-old adults to 38% for adults 70 years and older.

Limited efficacy is not unique for the zoster vaccine but shared with other vaccinations in older adults, including the annual influenza vaccine. To develop a non-empiric, mechanistic approach to improve the success of vaccination, recent studies of several vaccines including yellow fever, live-attenuated influenza virus (LAIV), inactivated influenza virus and two meningococcal polysaccharide vaccines have tried to identify system networks that orchestrate the vaccine response and can be targeted [6]. For influenza vaccination-induced responses, predictive models were developed based on cell population frequencies alone [7]. In many of these studies, transcriptional blood profiles early after vaccination were most informative and correlated with vaccine-induced antibody responses [8–16].

The underlying causes of VZV reactivation that zoster vaccination is supposed to mitigate are related to defects in cellular and not to those in humoral immunity. The strong correlation of herpes zoster with increasing age has been linked to a decrease in the frequency of VZV-specific T cells [17]. In contrast, antibody levels do not decrease with age and are not sufficient to prevent reactivation after stem cell transplantation [18]. Given the importance of cellular immunity in controlling infection, the objective of vaccination is to increase the frequency of VZV-specific long-lived memory T cells poised to produce IFN-γ, likely the best surrogate marker for protection against herpes zoster reactivation [19].

Antigen-specific T cells follow a typical sequence of events after vaccination or infection. First, they exponentially expand and differentiate into effector cells including follicular helper cells that control the activation of B cells and the generation of antibodies. While most of these effector cells undergo apoptosis, a small subset constitutes memory T cell precursors that differentiate into long-lived memory cells [20,21]. In addition to the extent of initial T cell expansion and help for B cell responses, success of vaccination therefore depends on effector T cells that escape cell death and survive as memory T cells. Elements of clonal expansion are affected by extrinsic factors such as antigen dose, strength and duration of stimulation and the initial inflammatory environment. Less is known about which cells survive contraction and by which mechanism. Initial stimulation strength appears to be important for the transition of CD4 effector into memory T cells [22,23]. Survival and transition into memory CD8^+^ T cells is supported by inhibition of mTORC1 activity [24]. However, systemic studies in particular for human vaccine responses have not been done.

Here, we assessed transcriptional profiles of whole blood and of isolated activated CD4 T cells and serum concentrations of cytokines in individuals receiving zoster vaccination to build predictive models of T cell responses. Inclusion of adults between the age of 50 and 79 years and nine twin pairs allowed determining the influence of age and genetic factors. Vaccine-induced increases in VZV-specific antibody titers and IFN-γ–producing T cells did not correlate. Frequencies of VZV-specific T cells peaked between day 7 and day 14 after vaccination. The subsequent contraction was the major determinant of long-lived VZV-specific T memory cell frequencies and correlated with gene expression modules in activated CD4 T cells.

## Results

### Influence of pre-existing immunity on vaccine responses

**Fig 1:**
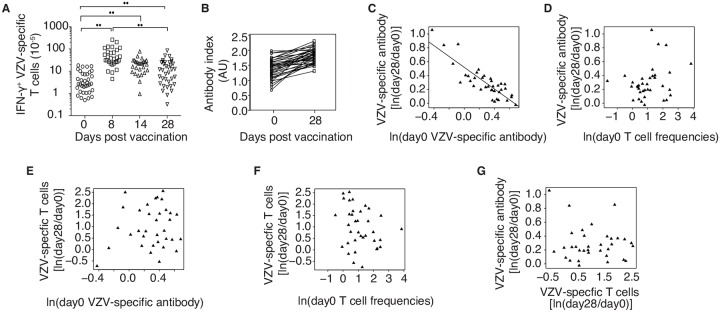
Influence of pre-existing VZV immunity on vaccine responses. (A) VZV-specific T cell frequencies were determined by IFN-γ–specific ELISpot before (day 0) and at days 8±1, 14±1 and 28±3 after vaccination. **p<0.0001 by paired Wilcoxon-Mann-Whitney test. (B) Antibody indices determined by VZV-IgG-specific ELISA increased between day 0 and 28 (p<0.01). Data from individual vaccinees are joined with a line. (C and D) Fold change in VZV-specific antibody concentrations from day 0 to day 28 were negatively correlated with initial antibody concentrations (C, r^2^ = 0.73, p <0.001), but not with initial VZV-specific T cell frequencies (D, p = 0.16). (E and F) Fold change in VZV-specific T cell frequencies from day 0 to day 28 showed no correlation with initial VZV-specific antibodies (E, p = 0.47) or initial T cell frequencies (F, p = 0.12). (G) Fold change in VZV-specific antibodies from day 0 to day 28 did not correlate with fold change in VZV-specific T cell frequencies from day 0 to day 28 (p = 0.38).

To identify host factors that influence the immunogenicity of the attenuated VZV pOka vaccine strain and the efficacy of VZV vaccination, we immunized 39 individuals aged 50 to 75 years, including 9 monozygotic twin pairs (Table 1). We measured VZV-specific T cell frequencies by IFN-γ–specific ELISpot, and VZV-specific antibody titers by ELISA. Experiments with PBMC depleted of either CD4^+^ or CD8^+^ T cells showed that the majority of VZV-specific T cells activated under these conditions were of the CD4^+^ T cell subset (S1 Fig). Frequencies of T cells producing IFN-γ in response to *in vitro* VZV lysate stimulation increased approximately 10-fold to peak between day 8 and 14 after vaccination and then declined (Fig 1A). By day 28, frequencies were on average three fold higher than before vaccination. Frequencies of global CD4 and CD8 populations did not change over the course of the vaccine response (S1 Fig). Concentrations of VZV-specific IgG antibodies consistently increased after vaccination although the increase was small in most individuals (median increase of 28%, 95% CI: 21%-41%, p<0.01) (Fig 1B).

**Table 1:** Demographics of study participants.

Subjects	Total	Twins	Average age	%ethnicity
Gender				
Males	20	6	62.7	95% Caucasian; 5% Hispanic
Females	19	12	60.6	85% Caucasian; 10% Hispanic; 5% unknown ethnicity
Age range				
50–59	19	16	55.9	90% Caucasian; 10% Hispanic
60–69	13	2	64.1	84% Caucasian; 8% Hispanic; 8% unknown ethnicity
70+	7	0	72.9	100% Caucasian

To determine whether the vaccine response was influenced by pre-existing VZV immunity, we ran linear regression analyses on the data (Fig 1C–1G). Consistent with previous literature, we found that VZV-specific T cell frequencies decline with age prior to vaccination (r = -0.46; p = 0.01), whereas antibody concentrations did not show significant correlation with age (p = 0.87). The magnitude of the antibody response (fold change from day 0 to day 28) inversely correlated with the initial antibody concentrations (p < 0.001, Fig 1C). A similar negative correlation has been observed with other vaccines and has been attributed to rapid clearance of the antigen by pre-existing antibodies [25]. Indeed, PCR for VZV DNA on days 1 to 3 in vaccinated individuals was always negative indicating the absence of viremia, despite the vaccine containing live virus. In contrast to antibodies, the magnitude of the T cell response (fold change in frequency from day 0 to day 28) showed no correlation with initial antibody concentrations suggesting that T cell responses are not influenced by pre-existing immunity (Fig 1E). Neither antibody nor T cell responses showed a correlation with initial T cell frequencies (Fig 1D & 1F). Correlation between increases in VZV-specific antibodies and T cell frequencies between day 0 and day 28 did not reach significance (p = 0.38, Fig 1G) indicating that these responses are relatively independent of each other.

### Accelerated loss of antigen-specific T cells in older individuals after vaccination

Following vaccination, the T cell response varied among individuals in terms of magnitude, but followed a consistent pattern, with frequencies of IFN-γ–producing VZV-reactive T cells peaking 8–14 days post-vaccination followed by a contraction in frequencies to a level that was higher than pre-vaccination frequencies. Using age alone to predict the increase in frequencies between day 0 and day 28 showed a negative trend, but did not reach significance (p = 0.18).

**Fig 2:**
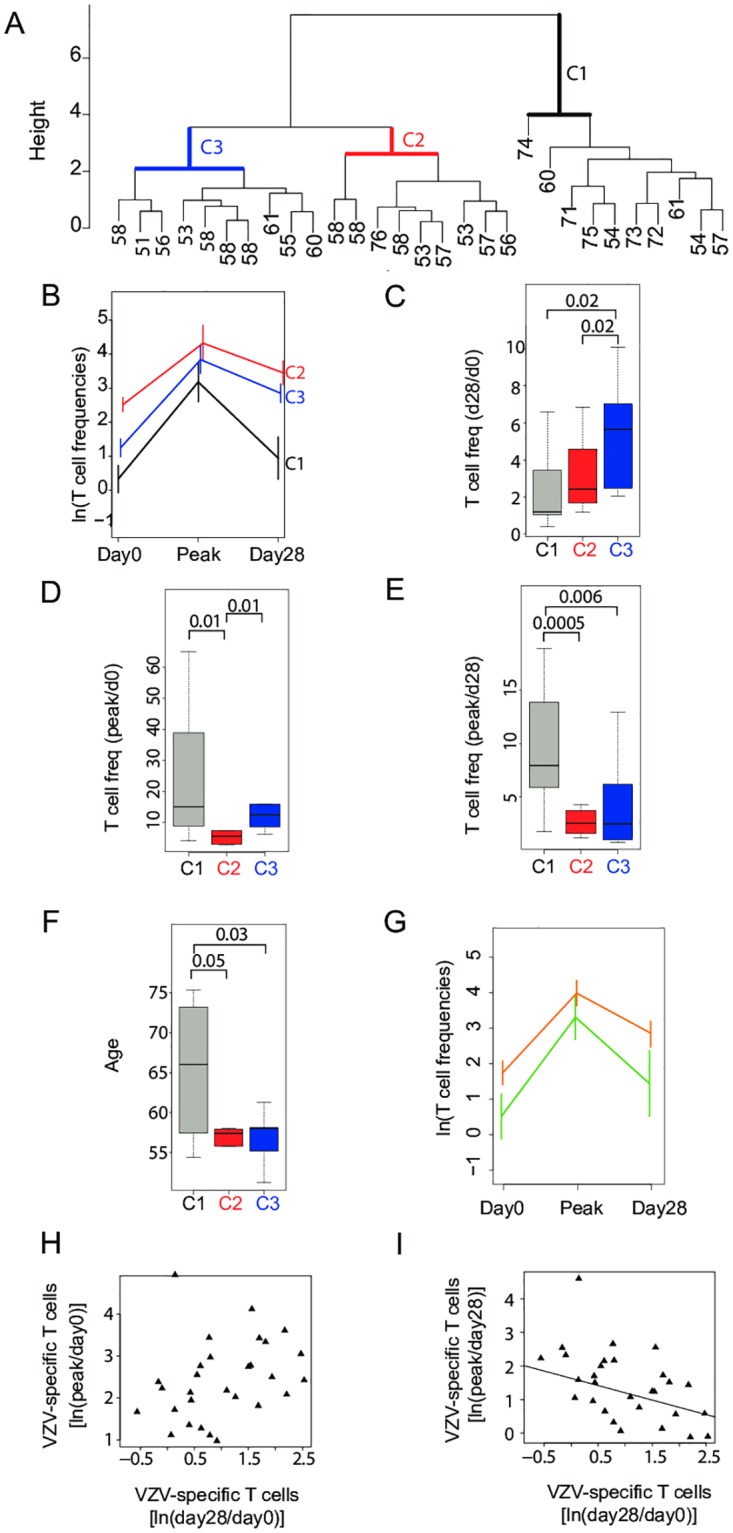
Contribution of initial expansion and subsequent contraction of VZV-specific T cells after vaccination to final T cell memory frequencies. VZV-specific T cell frequencies were measured by IFN-γ–specific ELISpot. Peak ELISpot counts were defined as the highest observed count at either day 8 (25/29 people) or day 14 (4/29 people) after vaccination. Absolute increase to the peak value (expansion) was calculated compared to day 0 counts. Absolute decline from peak (contraction) was calculated compared to day 28 counts. (A) Unsupervised hierarchical clustering analysis based on frequencies of VZV-specific T cells at all three time points. Vaccinees separated into three main clusters [C1 (black), C2 (red), C3 (blue)]. The color code for the different clusters is maintained in Fig 2B–2F. (B) Mean±SD of VZV-specific T cell frequencies at day 0, peak, and day 28 are shown for each cluster. (C-F) Boxplots showing overall increases in VZV-specific T cell frequencies from day 0 to day 28 (C), expansion in VZV-specific T cell frequencies from day 0 to peak value (D), contraction in VZV-specific T cell frequencies from peak to day 28 (E), and age range of individuals in each cluster (F). p-values calculated by one-tailed Wilcoxon-Mann-Whitney test are shown. (G) Vaccinees were grouped according to their age < 59 (orange) or >59 (green) years and T cell frequency trajectories after vaccinations are shown as described in Fig 2B. (H) When corrected for age, VZV-specific T cell expansion showed a weak correlation with overall increase in VZV-specific T cell frequency from day 0 to day 28 that did not reach significance (r = 0.31, p = 0.10). (I) Contraction after peak responses corrected for age inversely correlated with overall increase in VZV-specific T cell frequency from day 0 to day 28 (r = -0.53, p = 0.003).

To identify variables influencing the T cell response, we performed an unsupervised hierarchical clustering of frequencies of IFN-γ–producing VZV-specific T cells at day 0, at the peak response at day 8 or 14, and at day 28. Individuals segregated into three clusters (Fig 2A). Individuals of Cluster 1 started with relatively low frequencies, responded to vaccination with a strong effector cell expansion, but then had a more pronounced contraction phase than the individuals from the two other clusters (Fig 2B). In contrast, individuals from Cluster 2 had the highest pre-vaccination frequencies, but only produced a small expansion from day 0 to peak frequencies. As a consequence, individuals from cluster 1 and 2 both gained the smallest benefit from vaccination with only a twofold increase in VZV-specific T cell frequencies between day 0 and day 28 (Fig 2C), but for different reasons; failure of effector cell expansion for Cluster 2 (Fig 2D) and failure to develop long-lived memory for Cluster 1 (Fig 2E). Cluster 3 showed the largest fold increase in specific T cell frequencies owing to effective effector cell generation in the first phase of the vaccine response (Fig 2D), and less contraction in the second phase (Fig 2E).

When we compared the three clusters for demographic variables, we found that individuals in cluster 1 were older than those in clusters 2 and 3 (Fig 2F, S1 Table). Conversely, when vaccinees were grouped into younger and older than 59 years, concordance between cluster C and older individuals was significant (OR = 11.07, 95% CI: 1.52–114.33, p = 0.01). Moreover, the T-cell frequency post-vaccination trajectories by age group (Fig 2G) showed a high resemblance of those of three clusters shown in Fig 2B. The average T-cell response of older vaccinees (age>59) was similar to that in cluster 1, while the average T-cell response of younger study participants was more comparable to those in clusters 2 and 3. Gender and MHC class I or MHC class II types did not correlate with clustering. In particular, no variable was identified that distinguished individuals from clusters 2 and 3.

Our data suggest that a higher attrition of antigen-specific T cells after peak expansion accounts for the smaller overall vaccine responses observed in older individuals. To determine whether cell attrition generally is the critical determinant irrespective of age, we examined the relative contribution of expansion and contraction to the overall (day 28 to day 0) gain in antigen-specific memory T cells after correction for age. The correlation of the overall T cell response with expansion showed a trend that did not reach significance (r = 0.31, p = 0.10; Fig 2H), while it inversely correlated with contraction (r = -0.53, p = 0.003; Fig 2I).

### Genetic influence on vaccine-induced T cell responses

**Fig 3:**
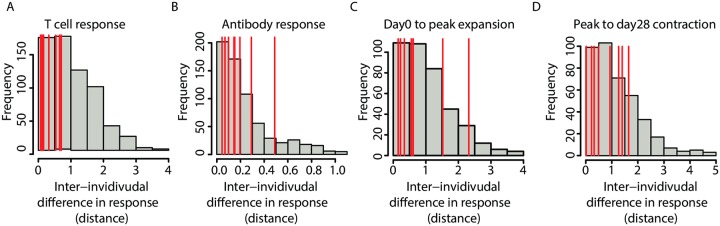
T cell responses to VZV vaccination are more similar in identical twins than in non-twins. Pairwise comparisons of fold changes in VZV-specific T cell frequencies (A, C, D) and antibodies (B) were performed for all individuals. Histograms show the frequency distributions of the differences in fold changes for each pair of individuals. Red lines show the position of identical twin comparisons. (A) T cell responses (day 28 to day 0) were more similar between identical twins than between unrelated individuals (p = 0.008). (B) Antibody responses were no more similar between twins than between unrelated individuals (p = 0.44). (C) Effector cell expansion (day 0 to peak) in twins was more similar compared with non-twins with two notable outliers, therefore not reaching significance (p = 0.28). (D) Contraction (peak to day 28) was slightly more similar between twins than between non-twins without reaching significance (p = 0.16).

Our cohort of vaccinees included 9 pairs of monozygotic twins. To examine the genetic influence on vaccine responses, we adjusted the response for age and performed pair-wise comparisons of participants. Each participant was compared with every other and the difference between each pair is plotted in Fig 3. The locations of pair-wise differences between identical twins are shown as red vertical lines.

In terms of the magnitude of the T cell response (fold change from day 0 to day 28), twin pairs were more similar than unrelated individuals; the average within-twin-pair difference was 36.5% of the average pair-wise difference between two unrelated individuals (p = 0.008; Fig 3A).

In terms of the magnitude of the antibody response (fold change from day 0 to day 28), identical twins were no more similar than unrelated individuals; the average within-twin-pair difference was 76.1% of the average pair-wise difference between two unrelated individuals (p = 0.44; Fig 3B), suggesting that there is little genetic influence on antibody generation following zoster vaccination.

When effector cell differentiation and contraction were assessed separately, the twin pairs only showed small trends toward similarity. The average within-twin-pair differences were 68.1% (expansion) and 69.6% (contraction) of the average pair-wise differences between two unrelated individuals. Either phase of the T cell response was not significantly more similar between twins than between unrelated individuals (p = 0.28 for expansion, Fig 3C; p = 0.16 for contraction Fig 3D). Thus, although the genetic make-up influences the generation of memory T cells, the accelerated loss after the peak response is mainly a function of age.

### Correlation of whole blood transcriptomes with vaccine responses

**Fig 4:**
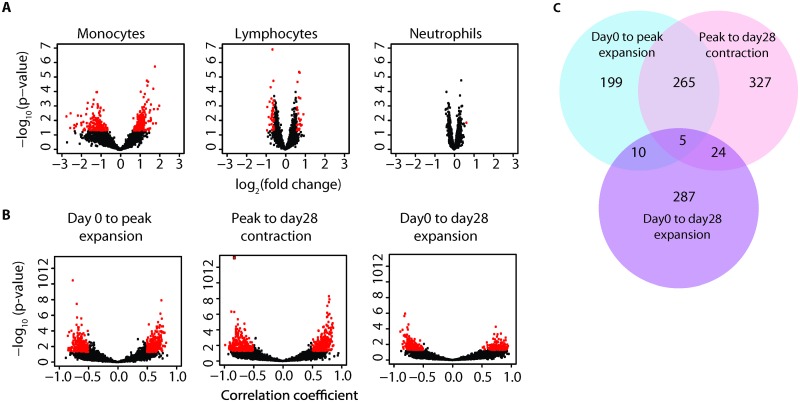
Correlation of whole blood-derived gene signatures with VZV-specific T cell responses. (A) Deconvolution of whole blood gene expression for leukocyte subsets was performed. Volcano plots show fold change of gene expression in monocytes (left), lymphocytes (middle) and neutrophils (right) between day 0 and day 1. The genes with significant changes in expression (fold change>1.5, p<0.05) after vaccination are colored in red. (B) Fold changes in monocyte-derived genes (day 0 to day 1, based on deconvolution analysis) were correlated with log-transformed changes in frequencies of antigen-specific T cells after VZV vaccination. Results are shown as volcano plots of correlation coefficients for VZV-specific T cell expansion (VZV-specific T cell frequencies day 0 to peak, left panel), contraction (peak to day 28, middle panel) and overall responses (day 28 to day 0, right panel). The genes with significant correlations (p<0.05) are colored in red. (C) The Venn diagram shows the overlap in genes that significantly correlate with expansion, contraction or global responses.

Gene expression arrays of whole blood were performed in vaccinees before (n = 28) and one (n = 18) or three days (n = 10) after vaccination. Cell-specific gene expression profiles were generated by deconvolution using previously described algorithms [26]. Given that zoster vaccine is a live vaccine, it was surprising that gene expression before and one or three days after vaccination were highly similar. Only very few neutrophil- and lymphocyte-related genes changed in expression from day 0 to 1 (Fig 4A). Significant changes for monocyte-related genes were found, but even here the number of probes with a significant change (n = 341 corresponding to 326 genes, p<0.05) was low after adjusting for false discovery (n = 9, FDR<0.1, S2 Table). When expression changes in monocyte-derived genes were analyzed for their correlation with T cell responses, we identified 493 probes corresponding to 479 genes that correlated with generation of VZV-specific effector T cells and 641 probes corresponding to 621 genes that correlated with the subsequent contraction phase with p<0.05 (Fig 4B, S3 Table). Interestingly, these two sets of genes were significantly overlapping (n = 268, p<0.0001), i.e., the same changes that were positively (n = 148) or negatively (n = 120) correlated with expansion inversely predicted contraction (Fig 4C); their effects therefore cancelled out in determining net benefit in memory cell generation. Pathways associated with the overlapping genes centered on TNF-α and STAT3 (S2 Fig). These data suggest that genes associated with monocyte activation influenced the expansion as well as apoptosis of short-lived effector T cells.

### Predicting T cell responses by vaccination-induced changes in serum cytokine concentrations

To assess other potential predictors of vaccine responses, we quantified serum cytokine concentrations before and after vaccination using 51-plex fluorescent bead assay. Again, for the first 10 participants, serum was assessed on days 0 and 3, for the subsequent 20 participants on days 0 and 1. Changes in serum cytokine levels were small and variable. Most changes on day 1 were no longer evident on day 3, therefore only changes from day 0 to day 1 were used in subsequent analyses.

**Fig 5:**
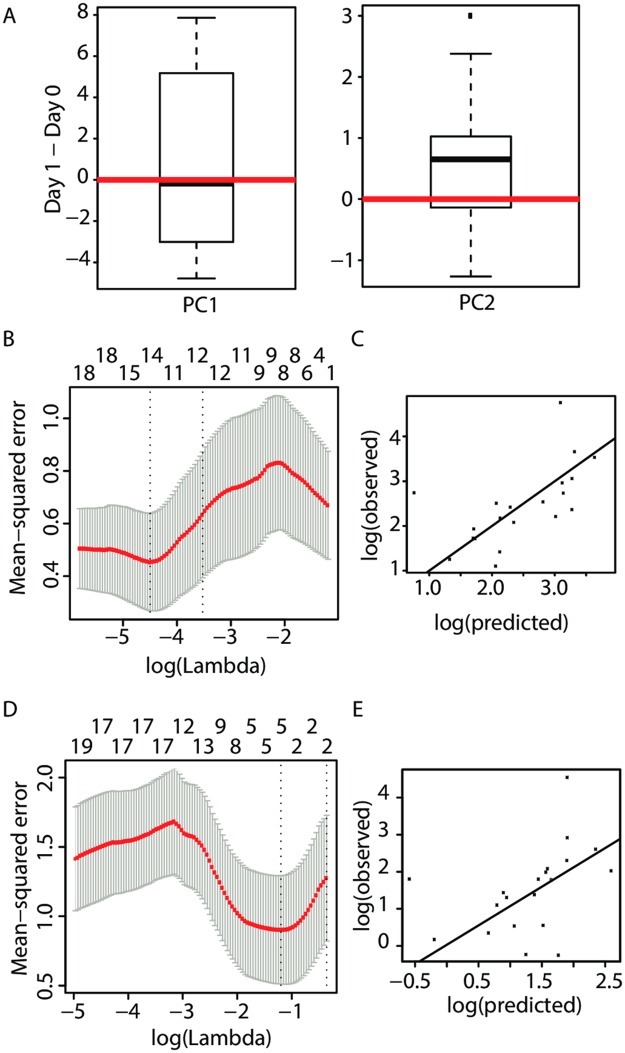
Lasso cytokine predictors of antigen-specific T cell expansion and contraction after VZV vaccination. (A) Principal component analysis of serum concentrations of 51 cytokines on day 0 and day 1 was performed. The box plots show the log transformed fold changes (day 1 to day 0) of PC1 (explaining 60% of variation, left panel) and PC2 (9%, right panel) among individuals. PC2 significantly changes between before and day 1 after vaccination (p = 0.017). (B-E) Panels B and D show the estimated mean square error (y-axis) from a sequence of lasso models in predicting VZV-specific T cell expansion (B) and contraction (D) using the baseline frequencies of VZV-specific T cells and serum cytokine changes between day 0 and day 1 after vaccination. The x-axis represents the log-transformed penalty parameter controlling the model complexity determined by the number of predictors in the model shown on top. Panels C and E plot the predicted vs. the true frequencies after leave-one-out cross-validation for the lasso procedures for VZV-specific T cell expansion (C) and contraction (E). The 45° line is shown for orientation.

Only the serum concentration of resistin showed a significant increase (p = 0.006). Other inflammatory cytokines that would be expected to increase during a viral infection did not show a consistent pattern. In a principal component analysis, vaccination contributed to the variability captured by PC2 (p = 0.017), but not by PC1 (Fig 5A).

To determine whether serum cytokine concentrations or their vaccine-induced changes influenced the vaccine-induced T cell expansion or contraction, we fitted a linear regression with lasso regularization to the data and constructed predictive algorithms, where the penalty parameter was selected via cross-validation. Prediction algorithms were tested using leave-one-out prevalidation. We identified two different Lasso algorithms based on cytokine changes that predicted expansion and survival of antigen-specific T cells, respectively and that passed prevalidation. These two algorithms included different sets of cytokines consistent with the notion that different mechanisms are involved in determining the two phases of the T cell response (Fig 5B–5E). Absolute cytokine concentrations by themselves were not predictive nor did their inclusion improve the Lasso predictors based on vaccine-induced changes in cytokine concentrations.

The following algorithm yielded a correlation of 0.99 for predicting expansion compared to 0.54 for baseline frequencies alone:

$$\begin{array}{c} \begin{array}{l} 3.98-0.60*\text{log}\left(\text{baseline T cell frequency}\right)\;+1.63*\left(\Delta \text{Leptin}\right)-0.78*\left(\Delta \text{MIG}\right)+0.06*\left(\Delta \text{ENA}78\right)-\\ 0.11*\left(\Delta \text{IP-}10\right)+1.11*\left(\Delta \text{IL-}17\right)+1.29*\;\left(\Delta \text{RANTES}\right)-1.43*\left(\Delta \text{IFN-}\alpha \right)-1.86*\left(\Delta \text{FGF-basic}\right)-\\ \;1.70*\left(\Delta \text{TRAIL}\right)-3.42*\left(\Delta \text{Resistin}\right)+4.23*\;\left(\Delta \text{M-CSF}\right), \end{array} \end{array}$$

where “Δ” denotes the change in cytokine concentration from day 0 to day 1 (Fig 5B). Following pre-validation, the correlation of predicted and observed increase in T cell frequencies was 0.64 compared with a correlation of 0.37 using initial T cell frequencies alone (Fig 5C). Of note, several inflammatory cytokines had a negative influence on T cell expansion, most notably IFN-α and resistin, the latter showing a significant increase following vaccination in the univariate analysis.

For predicting contraction, changes in cytokine distribution yielded an algorithm that correlated with the observed magnitude of the contraction and continued to outperform prediction by baseline frequencies alone (0.42 vs 0.20) after prevalidation (Fig 5D and 5E). The algorithm for predicting contraction using changes in cytokine concentration was as follows:

2.00−0.41*log(baseline T cell frequency)+0.05* (ΔLeptin)−0.04*(ΔTGF-β) + 0.06*(ΔIFN-α)+2.12*(ΔIL-1RA),

where “Δ” denotes the change in cytokine concentration from day 0 to day 1.

While the Lasso predictor of effector cell expansion used 11 of 51 possible terms raising concerns of overfitting, the prediction model for contraction only included four cytokines. Moreover, three of four terms in the contraction model (Leptin, IFN-α, IL-1RA), also appeared in a Lasso model predicting the increase in memory cell frequency from day 0 to day 28, although the latter failing cross-validation. In contrast, only 2 out of the 11 terms in the day 0 to peak expansion model (IFN-α and resistin) contributed to the model predicting day 0 to day 28 increases in cell frequencies. These findings re-emphasize the importance of contraction in determining the increase in frequencies of long-lived memory T cells after VZV vaccination.

### Predicting generation of VZV-specific T memory cells by gene expression in activated CD4 T cells

**Fig 6:**
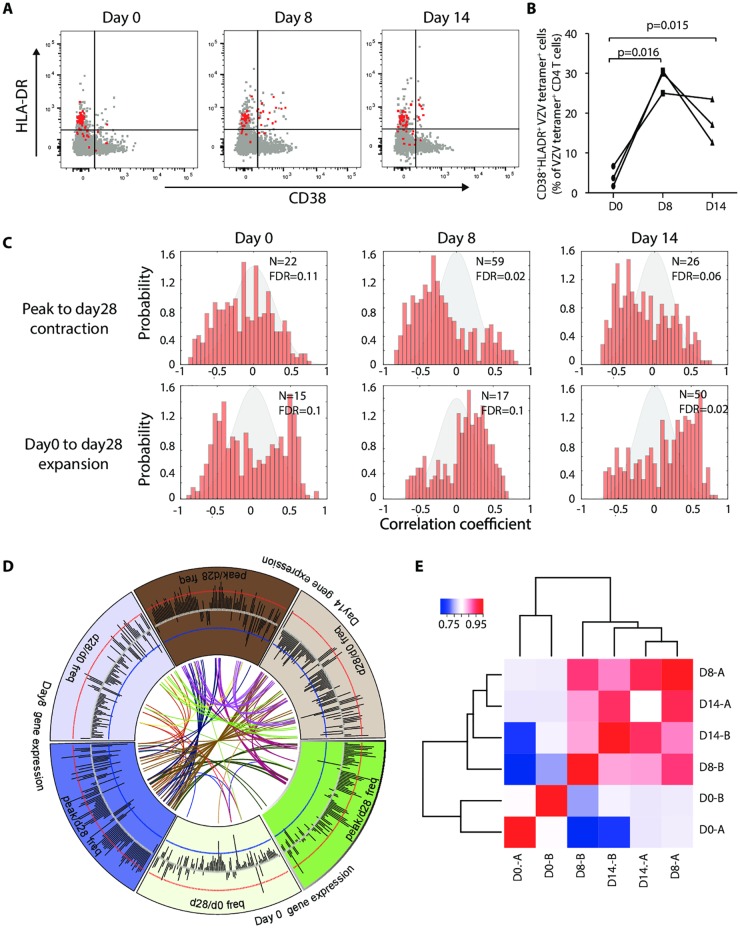
Correlation of gene signatures in activated CD4 T cells with VZV-specific T cell responses. (A) PBMCs of one donor before (day 0) and at days 8 and 14 after vaccination were gated on either total CD4 T cells (grey) or IE63 HLA-DRB1*15:01-tetramer-specific CD4 T cells (red). Expression of CD38 and HLA-DR is shown. (B) Frequencies of CD38^+^HLA-DR^+^ cells in total IE63 HLA-DRB1*15:01-tetramer-specific CD4 T cells of three individuals before (day 0) and on days 8 and 14 after vaccination. Time points are compared by paired t-test. (C) CD4^+^CD38+HLA-DR^+^ T cells were sorted before (day 0, left panels) and on days 8 (middle panels) and 14 (right panels) after vaccination and arrayed for gene expressions. Expression data were summarized as transcriptional modules and compared to changes in frequencies of VZV-specific T cells determined by IFN-γ ELISpot as shown in Fig 1. Histograms show probability distributions of Pearson correlation coefficients of expressed gene modules (red bars) with decline in VZV-specific T cell frequencies (peak to day 28, upper panels) and with overall increase in frequencies (day 0 to day 28, lower panels). Grey shaded curves represent probability distributions of correlations between random data generated from permutations of module gene labels and sample labels. FDR is calculated using linear regression analysis. The number of significantly associated modules is given as N for p<0.01. (D) The circos plot shows the sharing of significant modules obtained from activated T cells on days 0, 8 and 14. Grey bars show the log2 p-value of correlation between modules and T cell responses. Red and blue circles represent thresholds for modules with significant negative correlation and positive correlation to T cell responses, respectively (p<0.01). Curved lines in the center connect significant modules that are shared. (E) Heat maps show hierarchical clustering of correlation coefficients from the comparisons of gene modules with the fold decrease of peak to day 28 frequencies (A) and the fold increase of day 28 to day 0 frequencies (B). Directionality of correlation coefficients for peak to day 28 contraction was inversed to have equal biological directionality.

Our analysis so far suggests that the initial T cell response in the first two weeks after vaccination is less important for memory cell formation than the cell survival after peak responses, in particular in older individuals. Extrinsic factors including monocyte activation appear to be more important for effector cell differentiation than for memory formation. We hypothesized that cell-intrinsic properties in activated T cells can be identified that correlate with T cell survival and memory cell formation. We focused on CD4 T cells since the majority of IFN-γ T cells in the ELISpot assays were CD4 T cells (S1 Fig). In contrast to CD8 T cells, where HLA-A2 tetramers can be used to follow antigen-specific responses in about half of the population, human MHC class II molecules are too polymorph to monitor antigen-specific CD4 T cells using tetramers in a population study. For CD8 T cells, co-expression of HLA-DR and CD38 define activated T cells that include antigen-specific T cells at the time of the peak response after vaccination [27]. To determine whether the same phenotype defines activated CD4 T cells, we monitored the vaccine response using HLA-DR15 tetramers loaded with the VZV IE63 peptide 24 or gE peptide 54. Results of three HLA-DRB1*15 individuals before and on days 8 and 14 after stimulation are shown in Fig 6A and 6B. Virtually all VZV tetramer-positive CD4 T cells stained for HLA-DR even before vaccination. Expression of CD38 was gained in a subset of about 30% of antigen-specific CD4 T cells after vaccination, suggesting that co-expression of HLA-DR and CD38 is a good surrogate marker to monitor T cell activation in CD4 T cells.

CD4^+^HLA-DR^+^CD38^+^ T cells were sorted on days 8 and 14 in 17 of the original 39 individuals (8 from cluster 1 and 9 from clusters 2 and 3) and arrayed for gene expression. In 13 of the 17 individuals, we also obtained CD4^+^HLA-DR^+^CD38^+^ T cells prior to vaccination. Demographics and vaccination-induced increases in VZV-specific ELISpot frequencies of these 17 individuals are given in S4 Table. Expression data were correlated with the increase in ELISpot frequencies from day 0 to day 28 and with the decline from peak responses to day 28. We used the set of 334 blood transcription modules that have been previously annotated according to their biological functions and/or tissue-specific expression patterns [13]. Significantly associated modules are shown in S5 Table. Fig 6C shows the distribution of the Pearson correlation coefficients for the modules. For both day 8 and day 14 samples, the distributions were highly skewed with many modules negatively correlating with contraction after peak responses and positively with gain in VZV-specific memory cells from day 0 to day 28. This directionality is also illustrated in the circos plot in Fig 6D; the log2(p-value)s shown in the plot, generally higher for days 8 and day 14, signify preferentially negative correlation with contraction and positive correlation with long-term increase. Moreover, the network display in the circos plot shows that the same modules (p<0.01) are frequently associated with contraction and long-term increase on days 8 and 14. This high concordance is also supported by non-hierarchical clustering of correlation coefficients that is illustrated in the heat plot in Fig 6E. In this analysis, directionality of correlation coefficient for peak to day 28 contraction was inversed to have equal biological directionality as day 0 to day 28 increase. Association of day 8 and day 14 expression of gene modules for both outcomes co-clustered, while day 0 expression formed a separate cluster. A formal concordance statistics in S6 Table shows the pairwise correlation coefficients between the rho describing the correlation of module expression with outcome. S6B Table shows the concordance of p-values significant at 0.05 level measured by Cohen’s kappa.

The highest number of gene modules significantly associated with attrition (p<0.01) was found for the gene expression on days 8 and 14 (59 and 26 modules, respectively, Fig 6C). 50 modules on day 14 were identified that correlated with increase in VZV-specific T cells on day 28. Dominant themes in these modules were that of cell cycle and cell division and that of DNA repair and mismatch repair (S5 Table). One peculiarity was the finding of B cell-related modules exclusively on day 8 indicating contamination with B cell-derived transcripts although cell sorting was strictly gated on CD4 T cells after exclusion of duplets.

**Fig 7:**
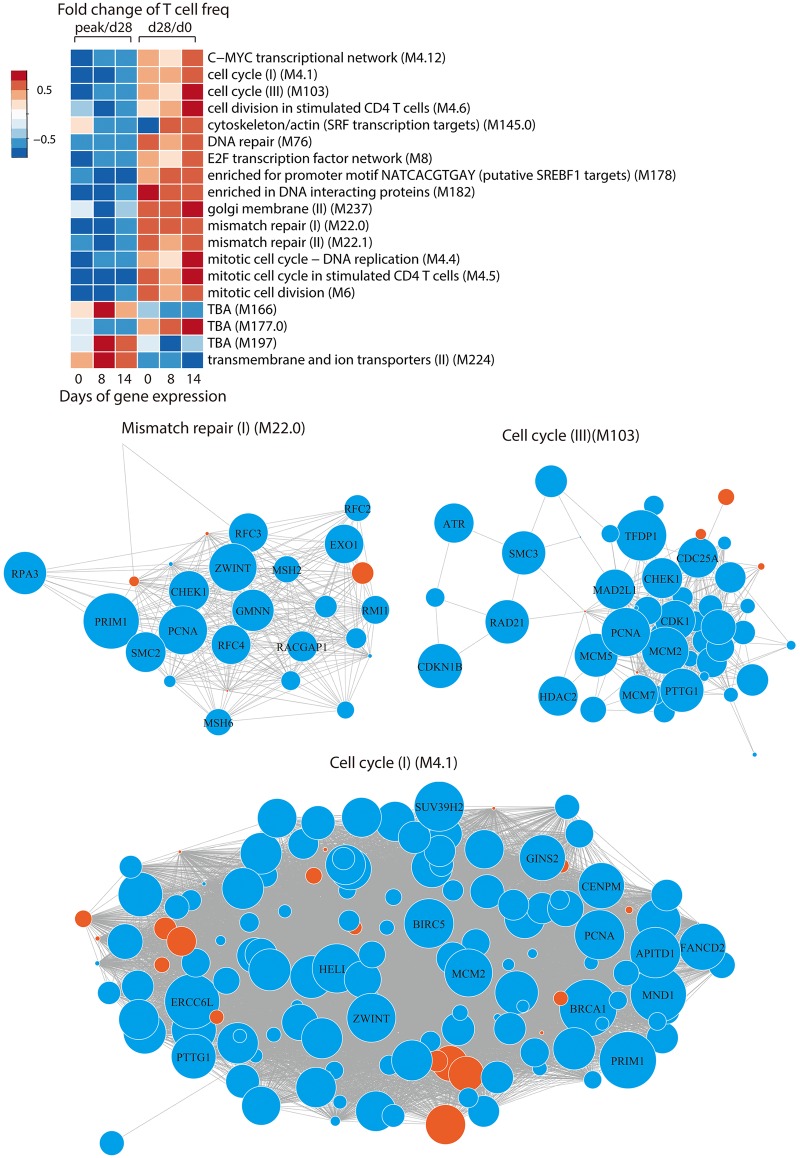
Preferential association of cell cycle and DNA repair pathways with VZV-specific T cell responses. (A) Correlation coefficients of modules that were significantly correlated with the decline in frequencies after peak responses as well as the overall increase from day 0 to day 28 as described in Fig 6, are shown as heat maps. Module nomenclature is from [13]. (B) Networks of genes in modules M22.0 (top left panel), M103 (top right panel) and M4.1 (bottom panel). The sizes of nodes represent the absolute value of correlation coefficients between gene expression level at day 14 after vaccination and peak to day 28 T cell responses. Orange and blue colors indicate positive and negative correlation, respectively. The grey line represents the co-expression relationship as described previously [13]. Gene names for the top genes with the highest correlation coefficient are listed.

As shown in Fig 6D, many of the significantly associated modules from days 8 and 14 arrays are shared and may be therefore particularly relevant. The heat plot in Fig 7A shows the correlation coefficients for those modules that were significantly associated with outcome at two time points and correlated with contraction of effector cells after peak responses as well as increase of long-term memory cells on day 28 compared to day 0. With the exception of a module of ion transporters and two undefined modules that were associated with increased contraction, expression of all of these modules correlated negatively with contraction and positively with successful generation of long-lived T memory cells.

As described in Fig 2, attrition was the critical step determining the increase in antigen-specific T cells after vaccination irrespective of age (Fig 2I), but was even more relevant in older individuals. The gene expression studies in activated CD4 T cells described in Figs 6 and 7 were done in a subgroup of 17 participants selected to include good and bad responders (S4 Table). The average age of these selected participants was lower than the original group with 60.6 years (inter-quartiles: 55.8–61.3) and only four individuals older than 70 years were included (S4 Table). Given the limited sample size, we only explored whether the modules shown in Fig 7A correlated with age. Correlation coefficients are shown as heat plot in S3 Fig. The directionalities of correlation coefficients were concordant for age and effector cell attrition and correlations reached a significance level of p<0.1 for seven modules in spite of the small sample. These data suggest that, while the identified gene modules account for higher attrition in general, they at least in part also explain the higher attrition with age.

The findings of the gene expression analysis suggested that regulation of cell division and DNA repair are critical processes determining effector cell survival and vaccination success. Three representative networks for day 14 are shown in Fig 7B, illustrating that most genes within one module contributed to the association and directionality of gene expression was highly uniform. In the cell cycle module M103, TFDP1 that dimerizes with E2F1 was highly correlated with attrition (r = -0.64) and d0 to d28 increase (r = 0.72) suggesting a role for G1-S1 transcriptional activation. In contrast, members of the E2F family were not associated. For gene module 4.1, BRCA1 had the highest association with attrition (r = -0.71), while Rad21, ATR, CDC25 and CHK1 genes in module M103 were significantly correlated, stressing the importance of DNA replication and DNA damage checkpoints [28]. Survivin or BIRC5, an anti-apoptotic molecule in the G2-M phase, was also negatively correlated with attrition as was the kinase CDK1 that functions as S and M phase regulator and phosphorylates survivin. Taken together, DNA structure checkpoints appear to be most important for the decision between apoptosis of effector cells and survival of long-lived memory cells.

## Discussion

Deciphering the molecular mechanisms that underlie the successful generation of immune memory after vaccination has remained a major challenge. Strategies to improve vaccine responses have so far been mostly empirical. To overcome these hurdles, systems biology approaches have been increasingly employed to identify pathways that influence the induction of vaccine-specific antibodies [8–15]. Here, we identify correlates of the induction of protective T cell immunity after vaccination with the live zoster vaccine. We found that the initial T cell expansion in the first days after vaccination and the subsequent cell death of effector T cells are at least in part independent processes contributing to the formation of long-lived memory T cells. Importantly, the extent of T cell contraction, determined by dysregulated cell cycle and DNA repair pathways, inversely correlated with memory cell generation and accounted for the compromised vaccine responses in older individuals.

T cell immunity is important for the control of latent or acute viral infections such as with VZV and influenza virus, respectively [29–31]. Determinants of protective T cell immunity are less defined than humoral immunity [32], though frequencies and repertoire breadth as well as polyfunctionality of antigen-specific T cells have been implicated [33,34]. In VZV vaccination studies in bone marrow recipients, the risk of zoster reactivation inversely correlated with the frequencies of VZV-specific CD4^+^ T cells [18]. In contrast, antibody titers did not correlate with reactivation. Similar to the T cell response to other herpes viruses, most of these CD4 T cells produce IFN-γ upon stimulation [35]. Consistent with previous studies, we found an age-associated decline in the frequencies of T cells that produced IFN-γ after *in vitro* restimulation with VZV while VZV-specific antibodies did not change [17]. It cannot be excluded that this decline reflects reduced ability to produce IFN-γ rather a decline in VZV-specific T cell frequencies; however, T cells *in vitro* expanded by VZV stimulation are mostly TH1 cells producing TNF-α and/or IFN-γ and only infrequently IL2 or TH2 cytokines [33]. We therefore decided to use the frequency of antigen-specific IFN-γ–producing T cells as a read-out for vaccination responses.

By including identical twins in our vaccination cohort, we were able to show a genetic influence on vaccine-induced T cell responses. Heritable factors for several vaccine responses have been shown for children [36,37]. In contrast, a recent study could not find such an association for the antibody response to influenza vaccination in the adult [38]. The authors discussed that immune parameters influenced by heritable factors early in life become more variable with age. Our study participants were older; still inherited influence reached significance for T cell responses although the study population was small. It is possible that B cell responses are more influenced by non-heritable factors; indeed, we did not find any influence of twin status on the induction of VZV-specific antibodies (Fig 3B and [39]).

Systems analysis in previous vaccine studies that used live viruses such as live-attenuated yellow fever or influenza viruses identified the activation of a number of pathways of the innate immune system that were important for the induction of antibody responses [8,9,13,14]. Innate immune activation was not very obvious with zoster vaccination; resistin, a member of the adipokine family, was the only inflammatory cytokine that significantly increased in the serum. Resistin is known to induce the expression of inflammatory cytokines; however, changes in the expression of inflammatory genes in peripheral blood were minor. Resistin’s role in adaptive immunity has not been extensively studied, also because of species differences: resistin is produced by adipocytes in mice and rats whereas by leukocytes in humans. Interestingly, it has been shown to inhibit dendritic cell function and thereby favor the induction of Tregs, which could explain its negative effect on T cell expansion after vaccination [40].

T cell responses after vaccination include a period of rapid expansion upon antigen stimulation followed by a contraction phase, during which most of the responding T cells die from apoptosis and do not transit into long-lived memory cells. Antigen-specific T cell frequencies after zoster vaccination peaked at day 8. This is earlier than described for the primary vaccine response to live viruses such as yellow fever, consistent with the vaccine response to VZV being a recall response [41]. We found expansion and effector cell differentiation to be poor predictors for the increase in frequencies of long-lived VZV-reactive memory CD4^+^ T cells. Monocyte activation and production of inflammatory markers favored T effector cell expansion. However, this gain was lost for T memory cell formation because the same genes were also associated with increased attrition. Overall, innate immune activation was minimal with zoster vaccination; resistin was the only inflammatory cytokine that significantly increased in the serum, and changes in gene expression were minor, which may explain why the influence of inflammatory genes on T cell responses was low.

Surprisingly, T cell expansion was relatively independent of age. This finding was unexpected because most models of immunosenescence imply declining T cell responsiveness to stimulation with age and in particular reduced ability to proliferate due to telomeric erosion and expression of p16 [42–44].

The extent of contraction clearly determined the benefit gained from vaccination. Equally important, increased attrition accounted for the declining vaccine response to zoster vaccination with age. The factors that determine survival of T cells after peak responses and differentiation into long-lived memory cells are largely unknown. For CD4^+^ T cells, transition into memory cells is dependent on the strength of the initial TCR signal and only high affinity T cells survive [22,23]. At the same time, differentiation of CD4^+^ T cells has been shown to be substantially influenced by environmental cues [45]. Vaccine-induced changes in serum concentrations of cytokines yielded Lasso predictors that were different for the expansion and contraction, consistent with the notion that the two phases are regulated by different mechanisms. The prediction models for after-peak contraction and increase in antigen-specific T cell frequencies from day 0 to day 28 were highly inversely correlated (r = 0.7) emphasizing the importance of effector cell attrition for the long-term benefit of vaccination.

As least equal to environmental factors, T cell survival and effector cell attrition are determined by cell-intrinsic factors that have been only incompletely defined [22,23]. Gene expression analysis of activated CD4 T cells co-expressing CD38 and HLA-DR on days 8 and 14 identified several gene modules that correlated with the increase in frequencies of VZV-specific T cells on day 28. Very strikingly, many of the associated modules were functionally related to cell cycle control or DNA repair. Interestingly, classical pro- or anti-apoptotic molecules that have been implicated in regulating cell attrition in the transition from effector to memory cells, like Bcl-2, Bim or PUMA did not show up in this analysis. Also, typical markers of T cell senescence such as hTERT, p16 or CD57 did not correlate with the vaccination-induced increase in antigen-specific memory cells. CD85j, a negative regulatory receptor expressed on CD8 TEMRA cells with age [46], but not other TEMRA- related molecules, was found to correlate with attrition.

The finding of gene modules involved in the regulation of DNA replication and DNA damage checkpoints is not entirely surprising. Obviously, T cells exit from a stage of rapid proliferation at the time when frequencies peak. Proliferative responses require the integration of cell cycle progression and survival mechanisms with proper DNA replication and DNA repair. Cell cycle progression is monitored by checkpoints in the G1/S, intra-S and G2/M phases that respond to DNA damage and activate DNA repair mechanisms [47,48]. Our findings suggest that this process determines the extent of attrition of effector T cells and therefore the survival of long-lived memory cells. Since multiple related modules were implicated, it is unclear whether one particular checkpoint or a limited number of driver genes can be identified that account for the failure in cell cycle regulation and DNA repair and the associated cell death.

The correlation between gene modules and attrition was maintained after correction for age, albeit with slightly lower significance. Gene modules identified here are therefore important for the survival of long-lived memory T cells in general independent of age. However, implicated gene expression modules also trended to correlate with age, and correlation coefficients for the correlation with attrition and age were generally concordant (S3 Fig) suggesting that these processes may be more frequently dysregulated in older individuals accounting for the increased effector cell attrition with age.

CD38 and HLA-DR double-positive T cells include VZV peptide tetramer-positive cells only after vaccination, but they certainly also include specificities other than those for VZV. It is therefore possible that gene modules found to be correlated with formation of long-lived memory cells are common for CD4 T cell activation in general. However, many modules that were shared in day 8 and day 14 activated CD4 T cells were not found to be predictive in activated CD4 T cells harvested before vaccination (Fig 6D) suggesting that they reflect the population of VZV-specific T cells that were activated by vaccination.

Our study on T cell responses after vaccination with the live zoster vaccine provides a unique opportunity to understand the constraints of a suboptimal T cell response as it occurs in an at least partially immunocompromised older population. One important conclusion is that the generation of effector T cells and of long-lived memory T cells are governed by at least in part different rulesets. Of particular interest is the finding that the contraction phase after the peak response is of utmost importance in memory cell formation, especially in older individuals. Contraction is mostly regulated by cell-intrinsic gene expression patterns and only to a lesser degree influenced by serum cytokines in the early stage of the immune response. It is currently unclear whether adjuvants that are commonly explored to promote T cell activation and expansion of effector cells also influence T cell memory cell differentiation. Interventions may be needed that improve the survival of expanded T effector cell populations [49]. A modulation of the cell cycle pathways implicated here may be the mechanism underlying the recent empirical observation that rapalogs can improve influenza vaccine response in the elderly [50].

## Materials and Methods

### Study population

Healthy individuals between the age of 50 and 75 years (20 males and 19 females, mean age 61.7 years) who had no history of shingles in the last five years and no prior vaccination with Zostavax were included in the study. Nine pairs of monozygotic twins were recruited from the SRI twin registry [51]. GoldenGate genotyping (Illumina Inc.) was performed to determine zygosity by IGenix (Bainbridge Island). Twins were considered identical if DNA markers were above 99% identical. Participants were vaccinated with live-attenuated VZV vaccine Zostavax (Merck & Co. Inc.). Peripheral blood was collected on the day of vaccination (day 0, prior to vaccine delivery), on day 1 or 3, and on days 8±1, 14±1 and 28±3 after vaccination.

### Ethics statement

The studies were approved by the Stanford University Institutional Review Board, and all participants gave informed written consent.

### ELISpot measurements of VZV-specific IFN-γ producing cells

PBMCs were isolated by Ficoll gradient centrifugation. In selected experiments, PBMC depleted of CD4 or CD8 T cells using the Miltenyi autoMACS cell separator and magnetobeads were used. Four serial dilutions starting with one million cells/well were added to precoated IFN-γ–specific ELISpot plates (Mabtech). Cell lysate from mock-infected (control) or pOKa-infected melanoma cells was added at a concentration of 3 μg/ml total protein, and cells were cultured for 16 h. Plates were developed according to the manufacturer’s instructions and analyzed using Immunospot software (Cellular Technology Limited). Mean spot counts were calculated from the two serial dilutions that were closest to 50 spots per well, followed by subtracting the spots of uninfected melanoma cells. The average background in PBMC cultured with lysate from uninfected melanoma cells was 0.46 spots per 100,000 PBMCs.

### ELISA measurements of VZV-specific IgG

Serum was collected at days 0 and 28. VZV-specific antibodies were determined using a commercially available kit (Calbiotech). Antibody indices are given in arbitrary units.

### Luminex analysis of cytokines

Serum samples from days 0, day 1 or day 3 were stored at -80°C until processed. Human 51-plex fluorescent bead assays (Affymetrix) were performed in duplicates as published [38]. MFI data for cytokines were analyzed by regression for age and batch. The regression coefficient for batch was used to normalize the data for estimated batch effects.

### MHC Class II tetramers

HLA-DRB1*15:01 monomers with thrombin-cleavable tethered CLIP peptide and Jun/Fos dimerization domains were produced in insect cells and purified by FPLC. The protein was biotinylated, peptide exchanged with VZV gE peptide 54 (TSPLLRYAAWTGGLA) and VZV IE63 peptide 24 (QRAIERYAGAETAEY) and tetramerized using PE- and APC-labeled streptavidin, respectively. Residual unbound streptavidin and biotinylated DR15 protein was removed from the tetramer product with biotin agarose (Sigma) and streptavidin agarose (ThermoFisher).

Prior to tetramer staining, PBMCs were pre-treated with 50 nM dasatinib (CST) for 30 minutes at 37°C. Tetramer staining (0.5μg/ml for each peptide-exchanged monomer) was performed for one hour at room temperature with Fc block (BioLegend) in 100 μl final volume. During the last 30 minutes of tetramer staining, cells were stained with antibodies to CD38 FITC; CD19, CD8, and γδTCR PerCP-Cy5.5; CCR7 PE-Cy7; CD45RA APC-Cy7; HLA-DR Pacific Blue; CD3 BV605; CD4 BV650 (all BioLegend); and with live/dead aqua zombie. Samples were washed and data were collected using an LSRII flow cytometer (Becton Dickinson). Data analysis was carried out with FlowJo software (TreeStar) gating on single tetramer-positive cells.

### Microarray analysis of whole blood samples

On day 0 and day 1, whole blood samples were collected into PAXgene tubes (PreAnalytiX). Total RNA was extracted using PAX gene blood RNA kits (Qiagen), labeled and hybridized to the Illumina HumanHT-12 V4 expression BeadChips.

Microarray raw data were quantile normalized by using GeneSpring software (Agilent). Deconvolution analysis was performed using a mixed effect regression model as previously described [26]. The analysis is built on the assumption that the observed gene expression level in the whole blood is the average cell-type specific gene expression levels weighted by their corresponding cell-type frequencies. Cell type frequencies were estimated by deconvolution from cell type-specific probes [52]. To identify significantly changed genes between day 0 and day 1, deconvolution analysis was performed on the top 5000 genes with highest variability in expression. Genes with 1.5-fold or greater changes in expression and p< 0.05 were considered as significantly changed. For analyzing association of immune responses with cell type-specific gene expression, deconvolution regression analyses were performed with p< 0.05 as filtering criterion.

### Microarray analysis of CD38^+^HLA-DR^+^CD4^+^ activated T cells

CD4 T cells expressing CD38 and HLA-DR were purified on days 0, 8±1 and 14±1 using an Aria cell sorter. Total RNA was extracted using RNeasy plus micro kit (Qiagen). The quality and quantity of RNA were checked by a 2100 Bioanalyzer (Agilent Technologies). Samples with RNA integrity values above 6 were used for further steps. cDNA was amplified from total RNA using Ovation PicoSL WTA System V2 (Nugen), followed by labeling and hybridizing to the SurePrint G3 human gene expression 8x60k microarrays (Agilent Technologies).

Microarray raw data were quantile normalized using GeneSpring software. Module activity scores, statistical significance and Pearson correlation of modules to immune responses were calculated using the btm_tool program in Python with p<0.01 as filtering criterion [13]. The circos plot was generated using Circlize package in R [53]. Hierarchical clustering was performed based on correlation coefficients with the correlation coefficients between modules and day 0 to day 28 responses inverted. The networks showing gene coexpression relationships of individual modules were generated using Pajek software.

### Statistical analysis

The continuous variables were summarized with mean and standard error. The associations between continuous variables such as age and immune response were tested using linear regression analysis with and without adjustment of confounding factors. The similarity between twins was assessed by permutation test to compare the average within twin-pair distance with the corresponding distance between unrelated individuals. The prediction models for frequency expansion and contraction were constructed by employing lasso-regularized linear regression. The penalty parameters in lasso were selected via the leave-one-out cross-validation, and the final prediction performance was evaluated by comparing the predicted versus observed outcomes via the pre-validation procedure. The concordance among pairwise associations of gene module and changes in VZV-specific T cell frequencies were measured by the correlation between correlation coefficients of the corresponding associations as well as the kappa statistics for concordance in significant associations. For the latter metric, a kappa between 0.40 to 0.75 significantly different from zero indicated fair to good concordance.

## Supporting Information

S1 Table Age demographics of individuals with different patterns of T cell responses after vaccination. (DOCX)

S2 Table Monocyte-related gene probes that significantly change between before and one day after vaccination (p<0.05). (DOCX)

S3 Table Monocyte-related genes that are significantly correlated with T cell responses (p<0.05). (DOCX)

S4 Table Age and VZV-specific T cell responses of individuals analyzed for gene expression in activated CD4 T cells. (DOCX)

S5 Table Gene expression modules in activated CD4 T cells that are significantly correlated with T cell responses (p<0.01). (DOCX)

S6 Table Concordance of gene expression modules in predicting VZV-specific T cell responses. (DOCX)

S1 Fig Dominance of CD4 T cells in VZV-specific ELISpot assays. (A) CD4 or CD8 T cells were depleted of PBMCs using anti-CD4 or anti-CD8 magnetic beads and the autoMACS cell separator. For undepleted cells, PBMCs were run through the cell separator without adding magnetic beads. Purity of CD4 and CD8 T cell subpopulation was assessed by flow cytometry. (B) VZV-specific T cell frequencies were determined by IFN-γ–specific ELISpot. CD4-depleted or CD8-depleted PBMCs were compared to undepleted PBMCs using paired Wilcoxon-Manny-Whitney test. The results suggest that under these culture conditions only VZV-specific CD4 T cells were detected. (C) Over the time course of 28 days after Zostavax vaccination, frequencies of global CD4 and CD8 populations did not change. A representative example is shown.
(DOCX)

S2 Fig Top scoring network of monocyte-expressed genes that significantly correlated with T cell responses as shown in Fig 4 were identified using IPA software. Red and green nodes represent genes that positively or negatively correlated. (A) Network of genes for which the change in expression correlated with both expansion and contraction and therefore not with long-term outcome (see Venn diagram Fig 4B). (B) Network of genes informative of long-term responses.
(DOCX)

S3 Fig Age relationship of gene expression modules that significantly correlated with T cell responses. Gene expression modules that were significantly correlated with the decline in frequencies after peak responses as well as the overall increase from day 0 to day 28 (Fig 7A), were examined for their correlation with age of study participants (S4 Table). Correlation coefficients shown as heat map exhibited a high concordance with those correlating expression levels with T cell attrition (Fig 7A).
(DOCX)
