# Supplementary material for: Defective T Memory Cell Differentiation after Varicella Zoster Vaccination in Older Individuals
Source: PLoS Pathog. 2016 Oct 20;12(10):e1005892. doi: 10.1371/journal.ppat.1005892 (PMC5072604; doi:10.1371/journal.ppat.1005892)
Supplement: S1 Table — (DOCX) [file ppat.1005892.s001.docx]

**Supplemental Table 1: Age demographics of individuals with different patterns of T cell responses after vaccination (Figure 2).**

|  | Mean | Min | 1^st^ Quartile | Median | 3^rd^ Quartile | Max |
| --- | --- | --- | --- | --- | --- | --- |
| Cluster C1 | 65.2 | 54.4 | 58.1 | 66.0 | 72.9 | 75.3 |
| Cluster C2 | 58.3 | 52.5 | 55.8 | 57.4 | 57.9 | 75.7 |
| Cluster C3 | 56.9 | 51.2 | 55.4 | 58.0 | 58.1 | 61.3 |
