# Supplementary material for: Defective T Memory Cell Differentiation after Varicella Zoster Vaccination in Older Individuals
Source: PLoS Pathog. 2016 Oct 20;12(10):e1005892. doi: 10.1371/journal.ppat.1005892 (PMC5072604; doi:10.1371/journal.ppat.1005892)
Supplement: S2 Table — (DOCX) [file ppat.1005892.s002.docx]

**Supplemental Table 2: Monocyte-related gene probes significantly changing in expression between before and one day after vaccination (p<0.05).**

| **Probe ID** | **Fold change** | **p-value** | **FDR** |
| --- | --- | --- | --- |
| 4260022 | 3.417 | 0 | 0.009 |
| 6450703 | 2.567 | 0 | 0.04 |
| 5890528 | 2.689 | 0 | 0.032 |
| 7610468 | 2.612 | 0 | 0.044 |
| 6180402 | 3.226 | 0 | 0.058 |
| 1450674 | 2.069 | 0 | 0.078 |
| 3170678 | 0.439 | 0 | 0.071 |
| 4540600 | 0.431 | 0 | 0.063 |
| 670112 | 2.385 | 0 | 0.074 |
| 2710725 | 2.289 | 0 | 0.11 |
| 780170 | 0.391 | 0 | 0.15 |
| 4880521 | 2.719 | 0 | 0.138 |
| 2570110 | 2.563 | 0.001 | 0.179 |
| 4810615 | 0.316 | 0.001 | 0.196 |
| 3890553 | 0.385 | 0.001 | 0.228 |
| 2940537 | 0.449 | 0.001 | 0.214 |
| 580025 | 2.406 | 0.001 | 0.204 |
| 6280706 | 2.973 | 0.001 | 0.199 |
| 6280326 | 3.98 | 0.001 | 0.227 |
| 4230487 | 0.456 | 0.001 | 0.237 |
| 70180 | 0.369 | 0.001 | 0.243 |
| 4860309 | 2.315 | 0.001 | 0.238 |
| 510619 | 2.178 | 0.001 | 0.244 |
| 6100040 | 0.5 | 0.001 | 0.246 |
| 6550603 | 0.449 | 0.001 | 0.237 |
| 7380349 | 0.497 | 0.001 | 0.251 |
| 1260288 | 2.481 | 0.002 | 0.253 |
| 2640471 | 0.309 | 0.002 | 0.249 |
| 4290097 | 3.778 | 0.002 | 0.253 |
| 4120369 | 3.294 | 0.002 | 0.249 |
| 2810626 | 2.218 | 0.002 | 0.259 |
| 1170706 | 0.555 | 0.002 | 0.275 |
| 5310369 | 2.888 | 0.002 | 0.271 |
| 3370092 | 2.345 | 0.002 | 0.276 |
| 3610064 | 2.024 | 0.002 | 0.298 |
| 2650110 | 0.485 | 0.002 | 0.291 |
| 270747 | 2.336 | 0.002 | 0.291 |
| 4060494 | 2.014 | 0.003 | 0.302 |
| 4730095 | 0.481 | 0.003 | 0.3 |
| 3170286 | 2.328 | 0.003 | 0.301 |
| 940711 | 2.21 | 0.003 | 0.31 |
| 5720470 | 2.82 | 0.003 | 0.303 |
| 3890193 | 2.382 | 0.003 | 0.296 |
| 4210241 | 0.471 | 0.003 | 0.304 |
| 6660162 | 0.172 | 0.003 | 0.324 |
| 360187 | 0.381 | 0.003 | 0.329 |
| 630377 | 2.133 | 0.003 | 0.326 |
| 630215 | 0.299 | 0.004 | 0.35 |
| 3170601 | 1.853 | 0.004 | 0.358 |
| 6980601 | 2.462 | 0.004 | 0.361 |
| 1340600 | 0.48 | 0.004 | 0.369 |
| 2680128 | 2.032 | 0.004 | 0.364 |
| 2230541 | 2.251 | 0.004 | 0.361 |
| 4640402 | 0.223 | 0.005 | 0.383 |
| 6510053 | 2.038 | 0.005 | 0.382 |
| 2450398 | 2.152 | 0.005 | 0.38 |
| 6480095 | 0.397 | 0.005 | 0.375 |
| 4730086 | 0.496 | 0.005 | 0.371 |
| 3390397 | 0.25 | 0.005 | 0.371 |
| 5670431 | 0.392 | 0.005 | 0.378 |
| 1300730 | 2.256 | 0.005 | 0.376 |
| 4260754 | 1.896 | 0.005 | 0.37 |
| 2260349 | 0.148 | 0.005 | 0.378 |
| 5690066 | 2.028 | 0.006 | 0.386 |
| 6270193 | 1.842 | 0.006 | 0.383 |
| 4890463 | 2.44 | 0.006 | 0.398 |
| 3310463 | 1.969 | 0.006 | 0.395 |
| 5260612 | 0.387 | 0.006 | 0.39 |
| 5690382 | 2.232 | 0.006 | 0.402 |
| 6550673 | 2.105 | 0.006 | 0.409 |
| 2000438 | 2.02 | 0.006 | 0.406 |
| 1260162 | 1.909 | 0.006 | 0.404 |
| 2850400 | 2.194 | 0.007 | 0.4 |
| 5550253 | 0.375 | 0.007 | 0.4 |
| 2940739 | 2.267 | 0.007 | 0.399 |
| 1070367 | 0.419 | 0.007 | 0.405 |
| 520209 | 2.942 | 0.007 | 0.404 |
| 3610192 | 0.458 | 0.007 | 0.401 |
| 4040008 | 1.598 | 0.007 | 0.402 |
| 3460754 | 2.205 | 0.007 | 0.407 |
| 240025 | 0.449 | 0.007 | 0.404 |
| 2360390 | 0.352 | 0.007 | 0.41 |
| 4880689 | 2.045 | 0.008 | 0.408 |
| 270403 | 2.089 | 0.008 | 0.419 |
| 2810296 | 2.322 | 0.008 | 0.424 |
| 5720703 | 1.856 | 0.008 | 0.42 |
| 3800671 | 0.442 | 0.008 | 0.432 |
| 4780615 | 1.953 | 0.009 | 0.449 |
| 1500707 | 0.398 | 0.009 | 0.466 |
| 3460504 | 0.418 | 0.009 | 0.463 |
| 7560435 | 0.432 | 0.009 | 0.461 |
| 10504 | 2.974 | 0.009 | 0.458 |
| 6560441 | 2.189 | 0.01 | 0.459 |
| 6860132 | 0.387 | 0.01 | 0.463 |
| 4220184 | 0.486 | 0.01 | 0.459 |
| 2350563 | 2.156 | 0.01 | 0.47 |
| 2810632 | 0.445 | 0.01 | 0.466 |
| 2600537 | 2.328 | 0.01 | 0.463 |
| 1260348 | 0.419 | 0.01 | 0.46 |
| 70176 | 2.024 | 0.01 | 0.457 |
| 1770603 | 2 | 0.01 | 0.459 |
| 3610241 | 2.166 | 0.01 | 0.455 |
| 6980170 | 2.088 | 0.011 | 0.463 |
| 6650035 | 0.469 | 0.011 | 0.465 |
| 2190671 | 2.912 | 0.011 | 0.473 |
| 2650300 | 2.716 | 0.011 | 0.474 |
| 3170561 | 2.238 | 0.012 | 0.489 |
| 2600066 | 1.915 | 0.012 | 0.492 |
| 5810632 | 1.937 | 0.012 | 0.491 |
| 5080280 | 0.434 | 0.012 | 0.5 |
| 4780537 | 0.514 | 0.013 | 0.509 |
| 2450563 | 0.358 | 0.013 | 0.507 |
| 2480274 | 0.271 | 0.013 | 0.503 |
| 3290110 | 1.83 | 0.014 | 0.532 |
| 1240097 | 0.492 | 0.014 | 0.528 |
| 6290400 | 2.253 | 0.014 | 0.543 |
| 6980039 | 0.457 | 0.014 | 0.54 |
| 620136 | 0.454 | 0.014 | 0.539 |
| 3990368 | 2.376 | 0.014 | 0.535 |
| 1340358 | 0.375 | 0.015 | 0.544 |
| 7610259 | 1.909 | 0.015 | 0.548 |
| 3440669 | 0.251 | 0.015 | 0.545 |
| 6060731 | 2.482 | 0.015 | 0.542 |
| 1770273 | 0.305 | 0.015 | 0.55 |
| 3940026 | 0.394 | 0.015 | 0.551 |
| 4830682 | 2.129 | 0.016 | 0.558 |
| 2470364 | 2.048 | 0.016 | 0.554 |
| 2030142 | 3.55 | 0.016 | 0.552 |
| 3360615 | 3.194 | 0.016 | 0.563 |
| 7200315 | 0.336 | 0.016 | 0.568 |
| 4760433 | 0.448 | 0.017 | 0.566 |
| 3930255 | 0.564 | 0.017 | 0.571 |
| 670072 | 2.024 | 0.017 | 0.573 |
| 2320653 | 1.836 | 0.017 | 0.569 |
| 2810364 | 2.279 | 0.017 | 0.566 |
| 4810474 | 0.448 | 0.017 | 0.572 |
| 2230376 | 0.215 | 0.018 | 0.581 |
| 360731 | 1.857 | 0.018 | 0.582 |
| 3060612 | 0.418 | 0.018 | 0.579 |
| 7200240 | 0.467 | 0.018 | 0.578 |
| 7380626 | 0.485 | 0.018 | 0.577 |
| 5670372 | 1.956 | 0.018 | 0.576 |
| 6840129 | 0.5 | 0.019 | 0.585 |
| 4210544 | 0.409 | 0.019 | 0.585 |
| 6580553 | 0.472 | 0.02 | 0.613 |
| 6380333 | 2.312 | 0.02 | 0.614 |
| 650446 | 0.509 | 0.02 | 0.614 |
| 2470162 | 0.346 | 0.02 | 0.617 |
| 2850068 | 2.214 | 0.02 | 0.615 |
| 5420398 | 2.184 | 0.021 | 0.616 |
| 430164 | 0.55 | 0.021 | 0.619 |
| 70070 | 0.252 | 0.021 | 0.618 |
| 2060170 | 0.197 | 0.021 | 0.614 |
| 5860243 | 2.048 | 0.021 | 0.613 |
| 5130139 | 0.597 | 0.021 | 0.611 |
| 3390292 | 1.939 | 0.021 | 0.609 |
| 4570091 | 1.806 | 0.021 | 0.606 |
| 2810471 | 2.338 | 0.021 | 0.607 |
| 2710358 | 2.152 | 0.022 | 0.616 |
| 6420626 | 0.525 | 0.022 | 0.617 |
| 3520156 | 1.837 | 0.022 | 0.619 |
| 990358 | 0.514 | 0.022 | 0.617 |
| 5290500 | 1.907 | 0.022 | 0.619 |
| 2570035 | 1.786 | 0.023 | 0.636 |
| 5910445 | 0.386 | 0.024 | 0.651 |
| 2120156 | 2.119 | 0.024 | 0.648 |
| 6380445 | 2.165 | 0.024 | 0.644 |
| 1170440 | 0.452 | 0.024 | 0.643 |
| 620047 | 0.477 | 0.024 | 0.646 |
| 6330615 | 1.981 | 0.024 | 0.643 |
| 6560338 | 1.779 | 0.024 | 0.64 |
| 4230136 | 0.537 | 0.024 | 0.639 |
| 6940086 | 0.34 | 0.025 | 0.639 |
| 5260132 | 0.466 | 0.025 | 0.636 |
| 5360553 | 2.066 | 0.025 | 0.634 |
| 6130446 | 2.235 | 0.025 | 0.631 |
| 6580239 | 1.921 | 0.025 | 0.631 |
| 3180324 | 0.534 | 0.025 | 0.632 |
| 6860347 | 0.33 | 0.025 | 0.632 |
| 840551 | 0.207 | 0.025 | 0.633 |
| 4150309 | 1.998 | 0.026 | 0.635 |
| 6550382 | 0.438 | 0.026 | 0.634 |
| 10112 | 1.901 | 0.026 | 0.631 |
| 770730 | 2.27 | 0.026 | 0.628 |
| 6860193 | 2.032 | 0.026 | 0.629 |
| 990689 | 1.702 | 0.026 | 0.629 |
| 450762 | 0.31 | 0.026 | 0.629 |
| 1050369 | 1.677 | 0.026 | 0.628 |
| 4260019 | 1.83 | 0.026 | 0.625 |
| 6760762 | 1.973 | 0.026 | 0.625 |
| 3930670 | 0.512 | 0.026 | 0.622 |
| 6020564 | 1.808 | 0.026 | 0.619 |
| 5220187 | 1.824 | 0.026 | 0.616 |
| 1740682 | 1.868 | 0.027 | 0.615 |
| 270133 | 0.437 | 0.027 | 0.612 |
| 1240064 | 0.457 | 0.027 | 0.61 |
| 2680100 | 0.373 | 0.027 | 0.61 |
| 5870184 | 0.516 | 0.027 | 0.615 |
| 770689 | 0.533 | 0.027 | 0.619 |
| 830326 | 0.486 | 0.028 | 0.618 |
| 7650097 | 0.439 | 0.028 | 0.62 |
| 3140056 | 1.818 | 0.028 | 0.618 |
| 6280167 | 0.593 | 0.028 | 0.62 |
| 4900440 | 1.73 | 0.028 | 0.617 |
| 4490520 | 1.916 | 0.028 | 0.615 |
| 5310537 | 1.911 | 0.028 | 0.615 |
| 3390349 | 0.51 | 0.028 | 0.613 |
| 1090497 | 0.468 | 0.028 | 0.613 |
| 6290681 | 1.903 | 0.028 | 0.611 |
| 6110605 | 1.857 | 0.028 | 0.609 |
| 4150121 | 0.46 | 0.029 | 0.609 |
| 1770609 | 0.477 | 0.029 | 0.609 |
| 2350066 | 0.511 | 0.029 | 0.608 |
| 1850139 | 0.527 | 0.029 | 0.607 |
| 4390327 | 1.652 | 0.029 | 0.605 |
| 5720059 | 1.938 | 0.03 | 0.617 |
| 4880392 | 0.391 | 0.03 | 0.615 |
| 4890093 | 1.889 | 0.03 | 0.623 |
| 830673 | 0.515 | 0.031 | 0.631 |
| 5700056 | 0.496 | 0.031 | 0.63 |
| 6110168 | 2.051 | 0.031 | 0.628 |
| 520333 | 0.489 | 0.031 | 0.628 |
| 360373 | 0.392 | 0.031 | 0.63 |
| 5390010 | 0.552 | 0.031 | 0.63 |
| 670026 | 2.292 | 0.032 | 0.63 |
| 520523 | 0.353 | 0.032 | 0.628 |
| 1440259 | 1.79 | 0.032 | 0.631 |
| 3610215 | 0.602 | 0.032 | 0.629 |
| 4260112 | 0.512 | 0.032 | 0.628 |
| 4490594 | 0.407 | 0.032 | 0.625 |
| 3840228 | 0.527 | 0.032 | 0.625 |
| 1470470 | 0.437 | 0.033 | 0.629 |
| 2900204 | 0.561 | 0.033 | 0.629 |
| 1010682 | 1.752 | 0.033 | 0.632 |
| 3440164 | 0.17 | 0.033 | 0.632 |
| 4670750 | 0.633 | 0.033 | 0.63 |
| 4390315 | 2.244 | 0.033 | 0.634 |
| 270156 | 1.765 | 0.034 | 0.642 |
| 160537 | 0.613 | 0.034 | 0.645 |
| 2900328 | 0.196 | 0.034 | 0.643 |
| 770561 | 0.403 | 0.034 | 0.641 |
| 1710630 | 1.702 | 0.035 | 0.64 |
| 2320114 | 2.113 | 0.035 | 0.64 |
| 2100332 | 1.815 | 0.035 | 0.639 |
| 7320370 | 1.821 | 0.035 | 0.636 |
| 4010181 | 0.405 | 0.035 | 0.634 |
| 5270110 | 1.81 | 0.035 | 0.632 |
| 430100 | 2.08 | 0.035 | 0.635 |
| 3420519 | 0.336 | 0.035 | 0.637 |
| 5550133 | 2.16 | 0.035 | 0.636 |
| 4480504 | 2.061 | 0.036 | 0.635 |
| 1260086 | 2.887 | 0.036 | 0.634 |
| 4280088 | 0.525 | 0.036 | 0.632 |
| 4180278 | 1.895 | 0.036 | 0.63 |
| 6550092 | 1.895 | 0.036 | 0.628 |
| 360056 | 1.795 | 0.036 | 0.627 |
| 7330253 | 1.787 | 0.036 | 0.626 |
| 2340739 | 2.808 | 0.036 | 0.626 |
| 4900670 | 1.849 | 0.037 | 0.642 |
| 3120136 | 0.511 | 0.037 | 0.64 |
| 1190220 | 1.649 | 0.038 | 0.645 |
| 4060333 | 1.81 | 0.038 | 0.644 |
| 830047 | 0.464 | 0.038 | 0.646 |
| 3190274 | 0.281 | 0.038 | 0.644 |
| 3120520 | 1.781 | 0.038 | 0.647 |
| 770424 | 1.981 | 0.038 | 0.647 |
| 6330338 | 0.525 | 0.038 | 0.647 |
| 5420367 | 1.978 | 0.039 | 0.647 |
| 4560021 | 0.327 | 0.039 | 0.646 |
| 2190403 | 1.733 | 0.039 | 0.647 |
| 2940022 | 1.903 | 0.039 | 0.646 |
| 4860327 | 2.051 | 0.039 | 0.647 |
| 7040280 | 2.642 | 0.039 | 0.645 |
| 870196 | 1.665 | 0.04 | 0.649 |
| 2000025 | 2.271 | 0.04 | 0.648 |
| 5050427 | 1.796 | 0.04 | 0.647 |
| 3060010 | 2.121 | 0.041 | 0.66 |
| 1990725 | 0.504 | 0.041 | 0.663 |
| 5810367 | 0.338 | 0.041 | 0.662 |
| 5820386 | 0.381 | 0.041 | 0.661 |
| 6100300 | 1.74 | 0.041 | 0.66 |
| 1470626 | 0.437 | 0.042 | 0.661 |
| 1850592 | 2.123 | 0.042 | 0.659 |
| 7100307 | 0.387 | 0.042 | 0.659 |
| 3460161 | 0.508 | 0.042 | 0.657 |
| 1190138 | 2.288 | 0.042 | 0.659 |
| 3180446 | 1.632 | 0.042 | 0.66 |
| 3370300 | 0.338 | 0.043 | 0.672 |
| 6840468 | 0.407 | 0.043 | 0.673 |
| 2360608 | 0.527 | 0.044 | 0.675 |
| 5890095 | 0.504 | 0.044 | 0.673 |
| 6960079 | 0.553 | 0.044 | 0.671 |
| 1940709 | 0.638 | 0.044 | 0.669 |
| 5960646 | 0.406 | 0.044 | 0.669 |
| 2100112 | 1.865 | 0.044 | 0.668 |
| 3140543 | 1.781 | 0.044 | 0.666 |
| 1190575 | 1.99 | 0.044 | 0.67 |
| 6520241 | 0.384 | 0.044 | 0.669 |
| 70634 | 0.458 | 0.045 | 0.67 |
| 1820632 | 0.494 | 0.045 | 0.668 |
| 6860300 | 1.751 | 0.045 | 0.669 |
| 540075 | 1.705 | 0.045 | 0.668 |
| 2120224 | 0.623 | 0.045 | 0.667 |
| 3460564 | 2.038 | 0.045 | 0.665 |
| 7210156 | 0.28 | 0.045 | 0.664 |
| 2810730 | 1.935 | 0.045 | 0.667 |
| 150224 | 0.484 | 0.046 | 0.668 |
| 7320041 | 0.429 | 0.046 | 0.666 |
| 5960747 | 2.421 | 0.046 | 0.666 |
| 7560053 | 0.566 | 0.046 | 0.666 |
| 1990487 | 0.552 | 0.046 | 0.664 |
| 5270730 | 2.019 | 0.046 | 0.662 |
| 2070209 | 1.96 | 0.046 | 0.662 |
| 1410221 | 0.287 | 0.046 | 0.66 |
| 2710523 | 0.542 | 0.046 | 0.661 |
| 3710544 | 1.898 | 0.047 | 0.661 |
| 3400646 | 0.526 | 0.047 | 0.659 |
| 1660296 | 2.415 | 0.047 | 0.658 |
| 2070288 | 0.525 | 0.047 | 0.656 |
| 6900279 | 0.387 | 0.047 | 0.654 |
| 5260008 | 0.416 | 0.047 | 0.656 |
| 6110474 | 0.52 | 0.047 | 0.655 |
| 10594 | 1.826 | 0.047 | 0.657 |
| 5570070 | 0.272 | 0.047 | 0.655 |
| 4390619 | 1.91 | 0.047 | 0.656 |
| 5390754 | 1.721 | 0.048 | 0.655 |
| 6450692 | 2 | 0.048 | 0.655 |
| 3450044 | 0.446 | 0.048 | 0.653 |
| 60719 | 0.354 | 0.048 | 0.652 |
| 2600131 | 0.596 | 0.048 | 0.651 |
| 5360079 | 2.29 | 0.048 | 0.65 |
| 620717 | 2.754 | 0.048 | 0.648 |
| 2750575 | 2.496 | 0.048 | 0.647 |
| 510228 | 1.779 | 0.048 | 0.651 |
| 6250280 | 1.72 | 0.048 | 0.649 |
| 4860538 | 1.722 | 0.049 | 0.649 |
| 5900682 | 1.989 | 0.049 | 0.647 |
| 1090102 | 1.622 | 0.049 | 0.648 |
| 1050367 | 0.538 | 0.049 | 0.649 |
| 110719 | 0.504 | 0.049 | 0.648 |
| 1940470 | 1.976 | 0.05 | 0.652 |
