# Supplementary material for: Defective T Memory Cell Differentiation after Varicella Zoster Vaccination in Older Individuals
Source: PLoS Pathog. 2016 Oct 20;12(10):e1005892. doi: 10.1371/journal.ppat.1005892 (PMC5072604; doi:10.1371/journal.ppat.1005892)
Supplement: S3 Table — (DOCX) [file ppat.1005892.s003.docx]

**Supplemental Table 3:** **Monocyte-related genes that significantly correlated with expansion (Day 0 to peak) or contraction (peak to day 28) of VZV-specific T cells (p<0.05)**

| **Genes correlating with expansion** | | | | **Genes correlating with contraction** | | | |
| --- | --- | --- | --- | --- | --- | --- | --- |
| **Gene name** | **FDR** | **p** | **rho** | **Gene name** | **FDR** | **p** | **rho** |
| ABHD5 | 0.041 | 0.001 | 0.689 | ABHD5 | 0.235 | 0.031 | 0.619 |
| ADD1 | 0.2485 | 0.032 | 0.5385 | ADAR | 0.225 | 0.029 | 0.745 |
| ADPRHL2 | 0.088 | 0.003 | 0.632 | ADD1 | 0.064 | 0.0015 | 0.7215 |
| AKIRIN2 | 0.196 | 0.019 | 0.534 | ADPRHL2 | 0.173 | 0.014 | 0.661 |
| ALDH2 | 0.023 | 0 | 0.702 | AKIRIN2 | 0.225 | 0.029 | 0.612 |
| ALOX5 | 0.074 | 0.002 | 0.751 | ALDH2 | 0.08 | 0.003 | 0.719 |
| ALOX5AP | 0.069 | 0.002 | 0.721 | ALOX5 | 0.177 | 0.015 | 0.747 |
| AMY2A | 0.109 | 0.005 | 0.611 | AMY2A | 0.207 | 0.023 | 0.636 |
| ANKRD35 | 0 | 0 | -0.763 | ANTXR2 | 0.172 | 0.014 | 0.689 |
| ANTXR2 | 0.198 | 0.019 | 0.629 | AP1M1 | 0.196 | 0.019 | 0.603 |
| AP1M1 | 0.054 | 0.001 | 0.587 | APEH | 0.076 | 0.003 | -0.661 |
| AP2S1 | 0.179 | 0.015 | -0.57 | APOBEC3G | 0.272 | 0.047 | -0.596 |
| APOBEC3D | 0.085 | 0.003 | -0.588 | ARAP3 | 0.113 | 0.006 | 0.704 |
| ARAP3 | 0.154 | 0.011 | 0.684 | ARF1 | 0.257 | 0.039 | 0.596 |
| ARHGAP25 | 0.182 | 0.016 | 0.526 | ARHGAP25 | 0.006 | 0 | 0.69 |
| ARHGAP30 | 0.125 | 0.007 | 0.646 | ARHGAP30 | 0.1685 | 0.024 | 0.6445 |
| ARHGDIB | 0.209 | 0.022 | 0.514 | ARHGAP9 | 0.169 | 0.014 | -0.717 |
| ARID4B | 0.02 | 0 | -0.581 | ARHGDIB | 0.268 | 0.043 | 0.592 |
| ARPC5 | 0.08 | 0.002 | 0.694 | ARHGEF1 | 0.195 | 0.018 | -0.74 |
| ASS1 | 0.313 | 0.049 | -0.537 | ARID1A | 0.241 | 0.034 | 0.61 |
| ATP5J | 0.12 | 0.006 | -0.645 | ARID4B | 0.084 | 0.003 | -0.623 |
| ATP8B2 | 0.298 | 0.044 | 0.538 | ARPC5 | 0.004 | 0 | 0.843 |
| B4GALT5 | 0.155 | 0.011 | 0.621 | ATP5H | 0.05 | 0.001 | -0.573 |
| B9D2 | 0.121 | 0.006 | 0.532 | ATP5J | 0.157 | 0.012 | -0.717 |
| BANP | 0.121 | 0.006 | 0.577 | ATP6V1B2 | 0.11 | 0.005 | 0.684 |
| BAT2D1 | 0.222 | 0.024 | 0.631 | B4GALT5 | 0.061 | 0.002 | 0.821 |
| BNIP3L | 0.121 | 0.006 | -0.561 | BANP | 0.065 | 0.002 | 0.689 |
| BOLA2 | 0.018 | 0 | -0.682 | BLZF1 | 0.236 | 0.032 | -0.908 |
| BTG1 | 0.231 | 0.026 | 0.601 | BNIP3L | 0.202 | 0.02 | 0.714 |
| BTN2A1 | 0.009 | 0 | 0.669 | BOLA2 | 0.012 | 0 | -0.797 |
| C10ORF54 | 0.006 | 0 | 0.666 | BTF3 | 0.112 | 0.005 | 0.807 |
| C12ORF35 | 0.136 | 0.008 | 0.77 | BTN2A1 | 0.036 | 0.001 | 0.725 |
| C12ORF57 | 0.055 | 0.001 | -0.652 | C10ORF54 | 0.219 | 0.027 | 0.585 |
| C14ORF4 | 0.028 | 0 | 0.738 | C12ORF35 | 0.244 | 0.035 | 0.799 |
| C1ORF152 | 0.042 | 0.001 | -0.747 | C12ORF57 | 0.025 | 0 | -0.79 |
| C1QBP | 0.207 | 0.02 | -0.535 | C16ORF13 | 0.035 | 0.001 | -0.622 |
| C2ORF28 | 0.063 | 0.002 | -0.535 | C17ORF61 | 0.172 | 0.014 | -0.635 |
| C5AR1 | 0.286 | 0.04 | 0.559 | C19ORF43 | 0.096 | 0.004 | -0.683 |
| C6ORF160 | 0.189 | 0.017 | -0.675 | C19ORF70 | 0.236 | 0.032 | -0.661 |
| C7ORF50 | 0.072 | 0.002 | -0.705 | C1ORF152 | 0.067 | 0.002 | -0.791 |
| CA1 | 0.293 | 0.042 | -0.682 | C1ORF24 | 0.227 | 0.029 | 0.722 |
| CAB39 | 0.252 | 0.031 | 0.507 | C1ORF55 | 0.194 | 0.018 | 0.602 |
| CALM2 | 0.285 | 0.04 | 0.536 | C1QBP | 0.193 | 0.018 | -0.659 |
| CALML4 | 0.284 | 0.039 | 0.575 | C20ORF43 | 0.119 | 0.006 | 0.557 |
| CAMK2G | 0.016 | 0 | 0.727 | C21ORF33 | 0.097 | 0.004 | 0.652 |
| CARD14 | 0.133 | 0.008 | -0.535 | C5ORF39 | 0.208 | 0.022 | -0.579 |
| CAST | 0.043 | 0.001 | 0.66 | C6ORF160 | 0.074 | 0.002 | -0.814 |
| CCDC72 | 0.266 | 0.034 | -0.693 | C7ORF50 | 0.255 | 0.039 | -0.661 |
| CCND3 | 0.058 | 0.001 | 0.644 | CAB39 | 0.151 | 0.011 | 0.645 |
| CCR2 | 0.15 | 0.01 | 0.607 | CALM2 | 0.193 | 0.018 | 0.666 |
| CD14 | 0.237 | 0.028 | 0.653 | CAMK2G | 0.194 | 0.018 | 0.653 |
| CD200 | 0.24 | 0.028 | -0.55 | CAMKK2 | 0.272 | 0.047 | -0.612 |
| CD52 | 0.165 | 0.012 | -0.711 | CAP1 | 0.135 | 0.008 | 0.58 |
| CD79B | 0.112 | 0.005 | -0.61 | CAPNS1 | 0.208 | 0.023 | 0.622 |
| CDC2L2 | 0.1545 | 0.014 | -0.535 | CARD14 | 0.045 | 0.001 | -0.731 |
| CDK5R1 | 0.04 | 0.001 | 0.609 | CASP1 | 0.193 | 0.018 | -0.736 |
| CENTB2 | 0.15 | 0.01 | 0.554 | CCDC147 | 0.095 | 0.004 | 0.581 |
| CHD1 | 0.27 | 0.034 | 0.605 | CCDC72 | 0.138 | 0.009 | -0.796 |
| CHES1 | 0.031 | 0 | 0.611 | CCDC93 | 0.262 | 0.041 | -0.597 |
| CHRNB3 | 0.29 | 0.041 | -0.529 | CCR2 | 0.199 | 0.0265 | 0.658 |
| CHST15 | 0.016 | 0 | 0.746 | CD14 | 0.079 | 0.003 | 0.773 |
| CITED2 | 0.12 | 0.006 | 0.653 | CD300A | 0.246 | 0.036 | 0.626 |
| CLC | 0.298 | 0.044 | -0.732 | CD300LF | 0.276 | 0.048 | 0.535 |
| CLEC16A | 0.085 | 0.003 | 0.613 | CD79B | 0.043 | 0.001 | -0.761 |
| CLEC2D | 0.307 | 0.047 | 0.518 | CD8A | 0.146 | 0.01 | -0.719 |
| CLTB | 0 | 0 | -0.695 | CD97 | 0.026 | 0 | 0.661 |
| CMIP | 0.015 | 0 | 0.646 | CDC2L5 | 0.208 | 0.023 | -0.523 |
| COMMD6 | 0.207 | 0.021 | -0.793 | CDC42 | 0.227 | 0.029 | 0.65 |
| COPZ1 | 0.196 | 0.019 | -0.525 | CDK2AP2 | 0.237 | 0.032 | -0.625 |
| COX6C | 0.277 | 0.036 | -0.701 | CENTB2 | 0.15 | 0.01 | 0.685 |
| CSF2RA | 0.143 | 0.009 | 0.645 | CHD1 | 0.224 | 0.028 | 0.698 |
| CTDSP1 | 0.062 | 0.001 | 0.566 | CHST15 | 0.146 | 0.01 | 0.71 |
| CTDSP2 | 0.086 | 0.003 | 0.653 | CITED2 | 0.153 | 0.011 | 0.704 |
| CTNNB1 | 0.169 | 0.013 | 0.541 | CLEC16A | 0.05 | 0.001 | 0.722 |
| CTSS | 0.04 | 0.001 | 0.74 | CLIC4 | 0.219 | 0.027 | 0.653 |
| CXCR4 | 0.097 | 0.004 | 0.648 | CLINT1 | 0.228 | 0.03 | 0.704 |
| CXORF38 | 0.208 | 0.022 | -0.511 | CLNS1A | 0.225 | 0.029 | 0.6 |
| CYSLTR1 | 0.022 | 0 | 0.702 | CLTB | 0.035 | 0.001 | -0.694 |
| DAB2 | 0.096 | 0.004 | -0.643 | CMIP | 0.001 | 0 | 0.753 |
| DDX3X | 0.195 | 0.018 | 0.553 | COMMD3 | 0.174 | 0.015 | -0.629 |
| DEM1 | 0.144 | 0.009 | 0.584 | COMMD6 | 0.206 | 0.022 | -0.862 |
| DENND5A | 0.176 | 0.014 | 0.618 | COX7B | 0.267 | 0.042 | -0.528 |
| DHRS9 | 0.201 | 0.02 | 0.72 | CPSF1 | 0.243 | 0.034 | 0.592 |
| DHX29 | 0.122 | 0.006 | -0.667 | CPVL | 0.234 | 0.031 | 0.729 |
| DHX38 | 0.139 | 0.009 | 0.569 | CREBBP | 0.266 | 0.043 | 0.62 |
| DPEP2 | 0.177 | 0.014 | 0.603 | CSDA | 0.134 | 0.008 | 0.758 |
| DPM2 | 0.275 | 0.035 | -0.584 | CTDSP2 | 0.007 | 0 | 0.784 |
| DPP9 | 0.307 | 0.047 | -0.502 | CTGLF3 | 0.256 | 0.039 | -0.656 |
| DPYD | 0.08 | 0.002 | 0.589 | CTNNA1 | 0.243 | 0.034 | 0.644 |
| DRAP1 | 0.101 | 0.004 | -0.602 | CUTA | 0.246 | 0.035 | -0.523 |
| DYRK2 | 0.29 | 0.041 | -0.592 | CXCR3 | 0.156 | 0.011 | -0.687 |
| ECGF1 | 0.013 | 0 | -0.666 | CYBA | 0.224 | 0.028 | 0.581 |
| EDG4 | 0.099 | 0.004 | 0.656 | CYBB | 0.216 | 0.026 | 0.571 |
| EEF1B2 | 0.206 | 0.021 | -0.715 | CYSLTR1 | 0.174 | 0.015 | 0.712 |
| EEF1D | 0.195 | 0.0185 | -0.585 | DAB2 | 0.01 | 0 | -0.798 |
| EFHD2 | 0.006 | 0 | 0.533 | DDX17 | 0.196 | 0.019 | -0.833 |
| EIF3L | 0.093 | 0.004 | 0.591 | DEM1 | 0.256 | 0.039 | 0.598 |
| EIF4G2 | 0.176 | 0.014 | 0.566 | DENND5A | 0.107 | 0.005 | 0.688 |
| EMR3 | 0.055 | 0.0015 | 0.814 | DERL1 | 0.016 | 0 | 0.72 |
| ERICH1 | 0.081 | 0.003 | 0.507 | DGCR14 | 0.086 | 0.003 | 0.535 |
| EVI2B | 0.305 | 0.046 | 0.518 | DIP2B | 0.252 | 0.037 | 0.578 |
| EVI5 | 0.277 | 0.036 | -0.668 | DNAJB6 | 0.144 | 0.01 | 0.725 |
| FADD | 0.282 | 0.038 | 0.549 | DPP9 | 0.28 | 0.05 | -0.655 |
| FAM39DP | 0.232 | 0.027 | -0.666 | DPYD | 0.246 | 0.035 | 0.565 |
| FAM49B | 0.142 | 0.009 | 0.722 | DRAP1 | 0.271 | 0.046 | -0.607 |
| FAM65A | 0.182 | 0.016 | 0.626 | DUSP1 | 0.142 | 0.009 | -0.98 |
| FAM8A1 | 0.192 | 0.018 | 0.504 | DUSP19 | 0.144 | 0.009 | -0.832 |
| FBXL10 | 0.244 | 0.029 | 0.535 | DUSP23 | 0.207 | 0.021 | -0.558 |
| FBXL15 | 0.18 | 0.015 | -0.557 | DYNLRB1 | 0.256 | 0.039 | -0.552 |
| FBXO33 | 0.13 | 0.007 | 0.551 | DYRK2 | 0.236 | 0.032 | -0.691 |
| FBXO7 | 0.034 | 0 | 0.726 | ECGF1 | 0.138 | 0.009 | -0.707 |
| FEZ2 | 0.266 | 0.033 | 0.585 | EDG4 | 0 | 0 | 0.788 |
| FGD3 | 0.015 | 0 | 0.682 | EEF1B2 | 0.082 | 0.003 | -0.838 |
| FKSG30 | 0.018 | 0 | 0.788 | EEF1D | 0.017 | 0 | -0.796 |
| FLI1 | 0.018 | 0 | 0.792 | EFTUD2 | 0.268 | 0.042 | 0.547 |
| FLJ38717 | 0.143 | 0.009 | -0.564 | EIF3L | 0.118 | 0.006 | 0.676 |
| FNBP1 | 0.207 | 0.021 | 0.573 | EIF3M | 0.267 | 0.043 | -0.673 |
| FNDC3B | 0.121 | 0.006 | 0.577 | EIF4A3 | 0.271 | 0.046 | 0.571 |
| FPR1 | 0.194 | 0.018 | 0.672 | EIF4E3 | 0.193 | 0.018 | 0.62 |
| FRAT1 | 0.034 | 0.001 | 0.6455 | ELF2 | 0.21 | 0.024 | 0.594 |
| FTHL12 | 0.283 | 0.038 | 0.637 | EMILIN2 | 0.224 | 0.028 | 0.655 |
| FTHL7 | 0.06 | 0.001 | 0.57 | EMR2 | 0.195 | 0.018 | 0.713 |
| FUCA2 | 0.194 | 0.018 | -0.531 | EMR3 | 0.159 | 0.012 | 0.852 |
| GAB2 | 0.313 | 0.049 | 0.524 | ERGIC1 | 0.218 | 0.026 | 0.734 |
| GABARAP | 0.207 | 0.021 | 0.611 | ERH | 0.036 | 0.001 | -0.702 |
| GALM | 0.06 | 0.001 | 0.502 | ERICH1 | 0.196 | 0.019 | 0.57 |
| GIMAP5 | 0.186 | 0.017 | -0.628 | FAM102A | 0.13 | 0.008 | -0.729 |
| GLB1 | 0.189 | 0.017 | 0.539 | FAM10A4 | 0.269 | 0.043 | 0.629 |
| GNL3L | 0.277 | 0.036 | -0.611 | FAM134A | 0.086 | 0.003 | -0.66 |
| GPR162 | 0.223 | 0.024 | 0.593 | FAM39DP | 0.206 | 0.021 | -0.612 |
| GPR177 | 0.305 | 0.046 | 0.616 | FAM65A | 0.193 | 0.018 | 0.728 |
| GPR56 | 0.101 | 0.004 | 0.612 | FAU | 0.241 | 0.034 | -0.533 |
| GPR65 | 0.184 | 0.016 | 0.526 | FBXL10 | 0.044 | 0.001 | 0.765 |
| GPX4 | 0.044 | 0.001 | -0.611 | FBXO33 | 0.195 | 0.018 | 0.605 |
| GTF2IP1 | 0.283 | 0.039 | 0.521 | FBXO7 | 0.038 | 0.001 | 0.836 |
| GTF3A | 0.037 | 0.001 | -0.612 | FCAR | 0.208 | 0.023 | -0.887 |
| GUSBL1 | 0.07 | 0.002 | -0.647 | FCGR3B | 0.018 | 0 | 0.758 |
| GZMA | 0.207 | 0.021 | -0.73 | FGR | 0.208 | 0.023 | 0.537 |
| HDAC7A | 0.023 | 0 | -0.651 | FLJ35801 | 0.194 | 0.017 | 0.637 |
| HERPUD1 | 0.304 | 0.046 | 0.51 | FLJ38717 | 0.27 | 0.045 | -0.676 |
| HINT1 | 0.259 | 0.032 | -0.7 | FNBP1 | 0.224 | 0.028 | 0.65 |
| HIST1H4C | 0.181 | 0.016 | -0.676 | FNDC3B | 0 | 0 | 0.796 |
| HIST2H2AA3 | 0.153 | 0.011 | -0.542 | FOS | 0.219 | 0.027 | 0.669 |
| HLA-B | 0.146 | 0.01 | 0.517 | FOXO3 | 0.207 | 0.023 | 0.622 |
| HLA-F | 0.059 | 0.001 | 0.573 | FRAT1 | 0.11 | 0.005 | 0.688 |
| HLA-H | 0.311 | 0.048 | 0.506 | FTHL12 | 0.149 | 0.01 | 0.793 |
| HMGCR | 0.085 | 0.003 | -0.543 | FTHL2 | 0.28 | 0.05 | 0.618 |
| HMGN1 | 0.093 | 0.004 | -0.647 | FTHL7 | 0.004 | 0 | 0.746 |
| HNRPH3 | 0.237 | 0.027 | 0.523 | FTHL8 | 0.176 | 0.015 | 0.756 |
| HS.143018 | 0.223 | 0.024 | -0.638 | FUT6 | 0.267 | 0.045 | -0.629 |
| HS.193767 | 0.182 | 0.016 | -0.809 | GAB2 | 0.181 | 0.016 | 0.684 |
| HS.413494 | 0.295 | 0.043 | 0.567 | GAB3 | 0.25 | 0.036 | 0.526 |
| HS.495041 | 0.093 | 0.004 | 0.628 | GABARAPL2 | 0.128 | 0.007 | 0.603 |
| HS.542428 | 0.172 | 0.013 | -0.583 | GALNT3 | 0.276 | 0.048 | -0.807 |
| HS.545976 | 0.091 | 0.003 | -0.766 | GAS7 | 0.234 | 0.031 | 0.613 |
| HS.569831 | 0.156 | 0.011 | -0.52 | GDI2 | 0.022 | 0 | 0.79 |
| HSPA1A | 0.154 | 0.011 | 0.716 | GDPD1 | 0.268 | 0.043 | -0.822 |
| HSPA6 | 0.103 | 0.0085 | 0.6795 | GIMAP8 | 0.266 | 0.043 | 0.547 |
| ICAM3 | 0.107 | 0.005 | 0.572 | GLB1 | 0.193 | 0.017 | 0.599 |
| IL1R2 | 0.272 | 0.034 | 0.762 | GNAI2 | 0.224 | 0.028 | 0.608 |
| IL8RB | 0.107 | 0.005 | 0.736 | GNG11 | 0.256 | 0.039 | -0.717 |
| ITGB2 | 0.129 | 0.0075 | 0.6365 | GNL3L | 0.206 | 0.021 | -0.682 |
| ITM2A | 0.028 | 0 | -0.674 | GPR177 | 0.19 | 0.017 | 0.728 |
| IWS1 | 0.085 | 0.003 | 0.621 | GRK5 | 0.114 | 0.006 | -0.631 |
| JAK1 | 0.208 | 0.022 | 0.621 | GRN | 0.134 | 0.008 | 0.725 |
| JARID2 | 0.119 | 0.006 | 0.534 | GTF2E2 | 0.142 | 0.009 | -0.509 |
| KDM3B | 0.021 | 0 | 0.631 | GTPBP4 | 0.249 | 0.036 | 0.583 |
| KIAA0174 | 0.277 | 0.036 | 0.513 | GUSBL1 | 0.006 | 0 | -0.793 |
| KIAA0319L | 0.196 | 0.019 | 0.543 | GZMA | 0.206 | 0.022 | -0.827 |
| KIAA1949 | 0.041 | 0.001 | 0.621 | HARS2 | 0.17 | 0.014 | 0.568 |
| KIF5B | 0.007 | 0 | 0.711 | HCFC1 | 0.196 | 0.019 | 0.672 |
| KLHL22 | 0.302 | 0.045 | 0.515 | HDAC1 | 0.214 | 0.025 | 0.517 |
| KLHL24 | 0.042 | 0.001 | 0.625 | HERPUD1 | 0.206 | 0.022 | 0.625 |
| KLRAQ1 | 0.058 | 0.001 | 0.591 | HHEX | 0.156 | 0.011 | 0.638 |
| L3MBTL2 | 0.222 | 0.024 | 0.521 | HLA-A29.1 | 0.008 | 0 | 0.86 |
| LAMP2 | 0.214 | 0.023 | 0.645 | HLA-B | 0.195 | 0.018 | 0.566 |
| LILRA5 | 0.139 | 0.009 | -0.565 | HLA-DRA | 0.268 | 0.043 | 0.623 |
| LILRB2 | 0.107 | 0.005 | 0.673 | HLA-DRB3 | 0.252 | 0.038 | 0.69 |
| LILRB3 | 0.13 | 0.008 | 0.658 | HLA-DRB6 | 0.001 | 0 | 0.762 |
| LMBRD1 | 0.207 | 0.021 | 0.657 | HMGB2 | 0.163 | 0.012 | 0.659 |
| LOC100128060 | 0.143 | 0.009 | -0.655 | HMGN1 | 0.279 | 0.049 | -0.643 |
| LOC100128936 | 0.035 | 0.001 | -0.622 | HNRPA2B1 | 0.151 | 0.01 | 0.652 |
| LOC100129034 | 0.295 | 0.043 | 0.597 | HS.149232 | 0.193 | 0.017 | 0.715 |
| LOC100129379 | 0.154 | 0.011 | -0.726 | HS.193767 | 0.154 | 0.011 | -0.846 |
| LOC100129650 | 0.231 | 0.026 | -0.584 | HS.208623 | 0.157 | 0.011 | -0.716 |
| LOC100129882 | 0.175 | 0.014 | -0.599 | HS.413494 | 0.218 | 0.026 | 0.701 |
| LOC100129902 | 0.024 | 0 | -0.75 | HS.520591 | 0.241 | 0.034 | -0.702 |
| LOC100130914 | 0.301 | 0.045 | 0.58 | HS.534061 | 0.195 | 0.018 | -0.746 |
| LOC100131166 | 0.102 | 0.004 | -0.51 | HS.538607 | 0.011 | 0 | -0.64 |
| LOC100131196 | 0.166 | 0.012 | -0.778 | HS.576106 | 0.199 | 0.02 | 0.688 |
| LOC100131387 | 0.248 | 0.03 | -0.84 | HS.580797 | 0.236 | 0.032 | -0.885 |
| LOC100131541 | 0.061 | 0.001 | -0.723 | HSPA1A | 0.209 | 0.024 | 0.714 |
| LOC100131971 | 0.067 | 0.002 | -0.752 | HTATIP2 | 0.169 | 0.014 | -0.59 |
| LOC100132037 | 0.096 | 0.004 | -0.752 | IFI27L2 | 0.247 | 0.035 | -0.616 |
| LOC100132266 | 0.119 | 0.006 | -0.576 | IFIH1 | 0.268 | 0.043 | 0.624 |
| LOC100132488 | 0.043 | 0.001 | -0.674 | IFP38 | 0.142 | 0.009 | -0.71 |
| LOC100132673 | 0.217 | 0.023 | -0.556 | IGF2BP2 | 0.234 | 0.031 | 0.834 |
| LOC100132715 | 0.306 | 0.047 | 0.538 | IK | 0.241 | 0.033 | 0.563 |
| LOC100134102 | 0.206 | 0.021 | -0.642 | IL8RB | 0.246 | 0.036 | 0.763 |
| LOC100134241 | 0.223 | 0.024 | 0.665 | IMAA | 0.235 | 0.031 | 0.555 |
| LOC100134273 | 0.131 | 0.007 | -0.576 | IRF2BP2 | 0.209 | 0.022 | 0.636 |
| LOC100134504 | 0.297 | 0.044 | -0.603 | ISY1 | 0.237 | 0.032 | -0.604 |
| LOC100170939 | 0.088 | 0.003 | -0.553 | ITGB1BP1 | 0.248 | 0.036 | 0.685 |
| LOC196752 | 0.131 | 0.008 | -0.542 | ITGB2 | 0.0855 | 0.0035 | 0.734 |
| LOC285900 | 0.179 | 0.015 | -0.655 | ITM2A | 0.022 | 0 | -0.778 |
| LOC347292 | 0.113 | 0.005 | -0.614 | IWS1 | 0 | 0 | 0.799 |
| LOC347544 | 0.207 | 0.021 | -0.508 | JAK1 | 0.234 | 0.031 | 0.686 |
| LOC387867 | 0.185 | 0.016 | -0.628 | JARID2 | 0.207 | 0.021 | 0.586 |
| LOC388275 | 0.037 | 0.001 | 0.645 | KCNH6 | 0.28 | 0.05 | -0.744 |
| LOC388339 | 0.128 | 0.007 | -0.746 | KDM3B | 0 | 0 | 0.758 |
| LOC388532 | 0.283 | 0.038 | -0.751 | KIAA0174 | 0.252 | 0.038 | 0.597 |
| LOC388564 | 0.204 | 0.02 | -0.559 | KIAA0907 | 0.227 | 0.029 | 0.588 |
| LOC389101 | 0.11 | 0.005 | 0.595 | KIAA1530 | 0.012 | 0 | 0.613 |
| LOC390414 | 0.001 | 0 | -0.618 | KIF5B | 0.236 | 0.032 | 0.592 |
| LOC391656 | 0.195 | 0.018 | -0.8 | KLHL28 | 0.189 | 0.017 | -0.896 |
| LOC391833 | 0.24 | 0.028 | -0.685 | KLRAQ1 | 0.218 | 0.026 | 0.536 |
| LOC401537 | 0.142 | 0.009 | -0.615 | KLRB1 | 0.113 | 0.006 | -0.761 |
| LOC402112 | 0.111 | 0.005 | -0.714 | KLRF1 | 0.209 | 0.023 | -0.702 |
| LOC402251 | 0.207 | 0.021 | -0.635 | L3MBTL2 | 0.107 | 0.005 | 0.639 |
| LOC440280 | 0.143 | 0.009 | 0.559 | LCP1 | 0.234 | 0.031 | 0.633 |
| LOC440311 | 0.02 | 0 | -0.641 | LILRA5 | 0.251 | 0.037 | -0.625 |
| LOC441506 | 0.075 | 0.002 | -0.636 | LILRA6 | 0.208 | 0.023 | 0.621 |
| LOC641814 | 0.168 | 0.013 | -0.573 | LIMS1 | 0.234 | 0.031 | 0.56 |
| LOC643287 | 0.022 | 0 | -0.744 | LMO2 | 0.24 | 0.033 | 0.648 |
| LOC644790 | 0.186 | 0.017 | -0.837 | LOC100128060 | 0.235 | 0.031 | -0.664 |
| LOC644863 | 0.067 | 0.002 | -0.68 | LOC100128196 | 0.276 | 0.048 | -0.556 |
| LOC644934 | 0.109 | 0.005 | -0.757 | LOC100128266 | 0.252 | 0.037 | -0.651 |
| LOC645251 | 0.003 | 0 | 0.69 | LOC100128936 | 0.08 | 0.003 | -0.776 |
| LOC646766 | 0.017 | 0 | -0.823 | LOC100129650 | 0.224 | 0.028 | -0.739 |
| LOC646849 | 0.286 | 0.04 | -0.519 | LOC100129657 | 0.096 | 0.004 | -0.721 |
| LOC646942 | 0.194 | 0.018 | -0.739 | LOC100129685 | 0.233 | 0.031 | -0.774 |
| LOC647099 | 0.153 | 0.01 | 0.565 | LOC100129758 | 0.12 | 0.007 | 0.591 |
| LOC647361 | 0.106 | 0.004 | -0.631 | LOC100129882 | 0.219 | 0.027 | -0.659 |
| LOC647910 | 0.13 | 0.007 | -0.518 | LOC100129902 | 0.225 | 0.029 | -0.774 |
| LOC648622 | 0.176 | 0.014 | -0.769 | LOC100130070 | 0.126 | 0.007 | -0.736 |
| LOC648771 | 0.305 | 0.046 | -0.506 | LOC100130178 | 0.169 | 0.013 | -0.547 |
| LOC649143 | 0.121 | 0.006 | -0.685 | LOC100130561 | 0.224 | 0.028 | -0.733 |
| LOC649661 | 0.259 | 0.032 | -0.534 | LOC100130604 | 0.239 | 0.033 | -0.525 |
| LOC650737 | 0.007 | 0 | -0.662 | LOC100130914 | 0.235 | 0.031 | 0.709 |
| LOC651309 | 0.132 | 0.008 | -0.507 | LOC100131166 | 0.066 | 0.002 | -0.656 |
| LOC652616 | 0.109 | 0.005 | 0.642 | LOC100131541 | 0.1135 | 0.0115 | -0.757 |
| LOC653316 | 0.023 | 0 | -0.605 | LOC100131971 | 0.122 | 0.007 | -0.82 |
| LOC653381 | 0.23 | 0.026 | 0.53 | LOC100132037 | 0.076 | 0.003 | -0.835 |
| LOC653778 | 0.252 | 0.031 | 0.648 | LOC100132457 | 0.268 | 0.043 | -0.617 |
| LOC653820 | 0.109 | 0.005 | -0.701 | LOC100132488 | 0.236 | 0.032 | -0.705 |
| LOC727761 | 0.136 | 0.008 | -0.566 | LOC100132673 | 0.21 | 0.024 | -0.656 |
| LOC727865 | 0.174 | 0.014 | -0.703 | LOC100132742 | 0.268 | 0.043 | -0.812 |
| LOC728553 | 0.194 | 0.018 | -0.67 | LOC100133005 | 0.079 | 0.003 | 0.689 |
| LOC728650 | 0.229 | 0.026 | 0.599 | LOC100133823 | 0.224 | 0.028 | -0.565 |
| LOC728666 | 0.248 | 0.03 | 0.631 | LOC100134102 | 0.28 | 0.05 | -0.659 |
| LOC728823 | 0.042 | 0.001 | -0.618 | LOC100134241 | 0.203 | 0.02 | 0.72 |
| LOC729009 | 0.315 | 0.05 | 0.553 | LOC100134273 | 0.216 | 0.025 | -0.648 |
| LOC729148 | 0.127 | 0.007 | 0.605 | LOC100134504 | 0.158 | 0.012 | -0.723 |
| LOC729236 | 0.015 | 0 | -0.785 | LOC134997 | 0.125 | 0.007 | -0.732 |
| LOC729340 | 0.298 | 0.044 | -0.687 | LOC196752 | 0 | 0 | -0.825 |
| LOC729500 | 0.044 | 0.001 | -0.641 | LOC284821 | 0.255 | 0.038 | -0.685 |
| LOC730029 | 0.203 | 0.02 | -0.509 | LOC285074 | 0.127 | 0.007 | 0.658 |
| LOC88523 | 0.005 | 0 | 0.721 | LOC285900 | 0.117 | 0.006 | -0.768 |
| LPIN2 | 0.033 | 0 | 0.606 | LOC338758 | 0.156 | 0.012 | -0.575 |
| LRG1 | 0.277 | 0.036 | 0.755 | LOC347376 | 0.005 | 0 | 0.769 |
| LRP10 | 0.28 | 0.037 | 0.577 | LOC347544 | 0.204 | 0.02 | -0.671 |
| LRRC25 | 0.208 | 0.022 | 0.655 | LOC374395 | 0.023 | 0 | 0.668 |
| LRRC47 | 0.153 | 0.011 | 0.563 | LOC388275 | 0 | 0 | 0.778 |
| LRRK2 | 0.277 | 0.036 | 0.652 | LOC388339 | 0.106 | 0.005 | -0.848 |
| LSP1 | 0.315 | 0.049 | 0.517 | LOC388532 | 0.144 | 0.01 | -0.858 |
| MAN2B2 | 0.081 | 0.003 | 0.591 | LOC388564 | 0.03 | 0.001 | -0.777 |
| MANSC1 | 0.007 | 0 | 0.776 | LOC388621 | 0.207 | 0.021 | -0.791 |
| MAP4K4 | 0.153 | 0.01 | 0.617 | LOC388789 | 0.227 | 0.029 | -0.545 |
| 6-Mar | 0.085 | 0.003 | -0.713 | LOC389156 | 0.218 | 0.026 | -0.832 |
| MBP | 0.21 | 0.022 | 0.649 | LOC389404 | 0.219 | 0.026 | -0.888 |
| MCOLN1 | 0.167 | 0.013 | -0.702 | LOC390414 | 0.16 | 0.012 | -0.511 |
| MED16 | 0.091 | 0.003 | 0.506 | LOC391045 | 0.271 | 0.046 | 0.691 |
| MED6 | 0.292 | 0.042 | 0.513 | LOC391656 | 0.271 | 0.046 | -0.855 |
| MFSD1 | 0.104 | 0.004 | 0.566 | LOC401152 | 0.016 | 0 | -0.705 |
| MGC3020 | 0.227 | 0.025 | -0.519 | LOC402112 | 0.097 | 0.004 | -0.794 |
| MGC33556 | 0.183 | 0.015 | -0.527 | LOC402175 | 0.184 | 0.016 | -0.601 |
| MGC72104 | 0.036 | 0.001 | -0.75 | LOC402251 | 0.268 | 0.044 | -0.689 |
| MIR1974 | 0.246 | 0.029 | -0.741 | LOC439953 | 0.199 | 0.02 | -0.534 |
| MKNK1 | 0.233 | 0.027 | 0.565 | LOC440926 | 0.196 | 0.019 | 0.537 |
| MLKL | 0 | 0 | 0.744 | LOC441246 | 0.279 | 0.049 | -0.745 |
| MME | 0.145 | 0.01 | 0.81 | LOC441506 | 0.168 | 0.013 | -0.635 |
| MMP25 | 0.14 | 0.009 | 0.796 | LOC641814 | 0.175 | 0.015 | -0.711 |
| MOBKL1B | 0.179 | 0.015 | 0.531 | LOC642250 | 0.165 | 0.013 | -0.9 |
| MPPE1 | 0.273 | 0.035 | 0.516 | LOC643287 | 0.142 | 0.009 | -0.838 |
| MRPL14 | 0.006 | 0 | -0.698 | LOC643977 | 0.173 | 0.014 | -0.591 |
| MRPL20 | 0.12 | 0.006 | -0.588 | LOC644790 | 0.224 | 0.028 | -0.884 |
| MRPL23 | 0.159 | 0.012 | -0.528 | LOC644863 | 0.023 | 0 | -0.798 |
| MRPL36 | 0.05 | 0.001 | -0.656 | LOC644907 | 0.124 | 0.007 | -0.69 |
| MRPL41 | 0.092 | 0.003 | -0.595 | LOC644934 | 0.206 | 0.022 | -0.856 |
| MRPL51 | 0.064 | 0.002 | -0.617 | LOC645251 | 0.066 | 0.002 | 0.693 |
| MRPL54 | 0.081 | 0.002 | -0.617 | LOC646766 | 0.108 | 0.005 | -0.849 |
| MRPS24 | 0.08 | 0.002 | -0.561 | LOC647030 | 0.28 | 0.049 | -0.729 |
| MRPS33 | 0.029 | 0 | -0.561 | LOC647285 | 0.268 | 0.045 | -0.671 |
| MSL3 | 0.112 | 0.005 | -0.614 | LOC647302 | 0.122 | 0.007 | -0.629 |
| MSL3L1 | 0.197 | 0.019 | -0.59 | LOC647340 | 0.273 | 0.047 | -0.608 |
| MST4 | 0.184 | 0.016 | 0.559 | LOC647361 | 0.237 | 0.032 | -0.712 |
| MT1E | 0.138 | 0.008 | -0.518 | LOC648622 | 0.272 | 0.047 | -0.815 |
| MTMR14 | 0.255 | 0.031 | 0.61 | LOC648705 | 0.205 | 0.02 | -0.662 |
| MTMR3 | 0.088 | 0.003 | 0.723 | LOC648771 | 0.207 | 0.023 | -0.512 |
| MTMR6 | 0.183 | 0.016 | 0.516 | LOC649821 | 0.249 | 0.036 | -0.781 |
| MXD1 | 0.187 | 0.017 | 0.705 | LOC650737 | 0 | 0 | -0.824 |
| MYADM | 0.314 | 0.049 | 0.598 | LOC651198 | 0.272 | 0.047 | -0.598 |
| MYH9 | 0.207 | 0.021 | 0.687 | LOC651309 | 0.105 | 0.005 | -0.705 |
| NAT5 | 0.104 | 0.004 | -0.581 | LOC653156 | 0.256 | 0.039 | -0.725 |
| NBPF20 | 0.081 | 0.003 | -0.65 | LOC653316 | 0.136 | 0.008 | -0.638 |
| NCF4 | 0.203 | 0.02 | 0.588 | LOC653381 | 0.195 | 0.018 | 0.638 |
| NCOA1 | 0.251 | 0.031 | 0.594 | LOC653505 | 0.125 | 0.007 | -0.631 |
| NCOA6 | 0.131 | 0.007 | 0.611 | LOC653778 | 0.082 | 0.003 | 0.838 |
| NDEL1 | 0.13 | 0.008 | 0.511 | LOC653820 | 0.114 | 0.006 | -0.779 |
| NDUFA1 | 0.119 | 0.006 | -0.574 | LOC654103 | 0.12 | 0.006 | 0.805 |
| NDUFA13 | 0.131 | 0.007 | -0.598 | LOC654194 | 0.147 | 0.01 | -0.894 |
| NDUFB2 | 0.081 | 0.003 | -0.67 | LOC727761 | 0.05 | 0.001 | -0.686 |
| NDUFB3 | 0.207 | 0.021 | -0.603 | LOC727826 | 0.113 | 0.006 | -0.683 |
| NFAT5 | 0.209 | 0.022 | -0.519 | LOC727865 | 0.051 | 0.001 | -0.844 |
| NFE2 | 0.098 | 0.004 | 0.611 | LOC728565 | 0.081 | 0.003 | -0.736 |
| NPL | 0.214 | 0.023 | 0.6705 | LOC728590 | 0.137 | 0.008 | -0.765 |
| NSUN5 | 0.133 | 0.008 | -0.517 | LOC728779 | 0.252 | 0.038 | -0.574 |
| NUDT1 | 0.083 | 0.003 | -0.55 | LOC728820 | 0.193 | 0.018 | -0.654 |
| OBFC2A | 0.081 | 0.002 | 0.663 | LOC728823 | 0.065 | 0.002 | -0.802 |
| OCIAD2 | 0.005 | 0 | -0.613 | LOC729009 | 0.074 | 0.002 | 0.743 |
| OSBPL2 | 0.278 | 0.036 | 0.598 | LOC729142 | 0.15 | 0.01 | -0.691 |
| PAM | 0.168 | 0.013 | 0.566 | LOC729148 | 0.194 | 0.018 | 0.587 |
| PANX2 | 0.166 | 0.012 | 0.69 | LOC729208 | 0.207 | 0.021 | -0.8 |
| PARP1 | 0.209 | 0.022 | 0.594 | LOC729236 | 0.071 | 0.002 | -0.836 |
| PASK | 0.091 | 0.003 | -0.585 | LOC729340 | 0.177 | 0.015 | -0.831 |
| PCBP1 | 0.184 | 0.016 | 0.672 | LOC729500 | 0.138 | 0.009 | -0.702 |
| PCYOX1 | 0.23 | 0.026 | -0.585 | LOC730029 | 0.224 | 0.028 | -0.663 |
| PDE7A | 0.119 | 0.006 | 0.535 | LOC730255 | 0.263 | 0.041 | -0.825 |
| PDLIM7 | 0.113 | 0.005 | 0.617 | LOC730740 | 0.156 | 0.011 | 0.722 |
| PECAM1 | 0.224 | 0.025 | 0.533 | LOC731789 | 0.214 | 0.025 | -0.781 |
| PELI1 | 0.154 | 0.011 | 0.638 | LOC731985 | 0.028 | 0 | -0.711 |
| PFN1 | 0.078 | 0.002 | 0.574 | LOC732007 | 0.193 | 0.017 | 0.661 |
| PGAM1 | 0.18 | 0.015 | 0.536 | LOC91561 | 0.25 | 0.037 | 0.587 |
| PGAM4 | 0 | 0 | 0.747 | LPAR2 | 0.276 | 0.048 | 0.723 |
| PGCP | 0.148 | 0.01 | 0.662 | LRP10 | 0.272 | 0.047 | 0.649 |
| PGD | 0.283 | 0.039 | 0.57 | LRPPRC | 0.118 | 0.006 | -0.606 |
| PGRMC2 | 0.143 | 0.009 | 0.567 | LRRK2 | 0.262 | 0.041 | 0.762 |
| PLCG2 | 0.234 | 0.027 | 0.614 | M6PR | 0.21 | 0.024 | -0.629 |
| PLEKHB2 | 0.001 | 0 | -0.69 | MAFF | 0.159 | 0.012 | -0.833 |
| PLOD1 | 0.184 | 0.016 | 0.6 | MAN2B2 | 0.006 | 0 | 0.702 |
| POGK | 0.152 | 0.011 | 0.574 | 6-Mar | 0.144 | 0.01 | -0.8 |
| PPT1 | 0.223 | 0.025 | 0.742 | MARCKS | 0.267 | 0.043 | 0.755 |
| PQBP1 | 0.14 | 0.009 | -0.547 | MBD4 | 0.224 | 0.028 | -0.875 |
| PRR13 | 0.054 | 0.001 | 0.702 | MBP | 0.094 | 0.004 | -0.55 |
| PRSS23 | 0.196 | 0.019 | 0.527 | MBTD1 | 0.225 | 0.029 | -0.816 |
| PSAP | 0.149 | 0.01 | 0.664 | MED16 | 0.063 | 0.002 | 0.657 |
| PSCDBP | 0.04 | 0.001 | 0.701 | MGC72104 | 0.056 | 0.001 | -0.835 |
| PSMA3 | 0.201 | 0.02 | -0.599 | MIF | 0.047 | 0.001 | -0.654 |
| PSMA4 | 0.172 | 0.013 | -0.592 | MLKL | 0.217 | 0.026 | 0.668 |
| PSMA6 | 0.08 | 0.002 | -0.579 | MMP25 | 0.246 | 0.035 | 0.816 |
| PSMC3 | 0.167 | 0.013 | -0.514 | MORF4L1 | 0.058 | 0.002 | 0.783 |
| PTOV1 | 0.306 | 0.046 | 0.535 | MRPL14 | 0.149 | 0.01 | -0.643 |
| PVALB | 0.183 | 0.015 | -0.555 | MRPL20 | 0.272 | 0.046 | -0.538 |
| QPCT | 0.314 | 0.049 | 0.654 | MRPL41 | 0.076 | 0.003 | -0.737 |
| RAB11FIP1 | 0.236 | 0.027 | 0.518 | MRPL51 | 0.012 | 0 | -0.744 |
| RAB27A | 0.071 | 0.002 | -0.578 | MRPL54 | 0.234 | 0.031 | -0.643 |
| RAB35 | 0.227 | 0.025 | 0.537 | MRPS33 | 0.096 | 0.004 | -0.656 |
| RAD23A | 0.223 | 0.024 | -0.711 | MSL3 | 0.04 | 0.001 | -0.801 |
| RAP1BL | 0.113 | 0.005 | 0.502 | MSL3L1 | 0.004 | 0 | -0.763 |
| RASSF2 | 0.168 | 0.013 | 0.561 | MT1E | 0.019 | 0 | -0.769 |
| RASSF5 | 0.2925 | 0.0415 | 0.512 | MTHFS | 0.142 | 0.009 | -0.622 |
| RB1CC1 | 0.044 | 0.001 | 0.681 | MTMR14 | 0.267 | 0.043 | 0.685 |
| RBED1 | 0.11 | 0.005 | -0.578 | MTMR3 | 0.003 | 0 | 0.853 |
| RBL2 | 0.019 | 0 | 0.755 | MYADM | 0.206 | 0.021 | 0.692 |
| RBM12B | 0.282 | 0.037 | -0.536 | MYH9 | 0.254 | 0.0395 | 0.695 |
| RBM33 | 0.083 | 0.003 | 0.593 | NAAA | 0.261 | 0.04 | 0.577 |
| RCBTB2 | 0.189 | 0.017 | 0.613 | NACA | 0.073 | 0.002 | 0.595 |
| REPS2 | 0.057 | 0.001 | 0.75 | NARF | 0.048 | 0.001 | 0.667 |
| RHOC | 0.105 | 0.004 | -0.582 | NAT5 | 0.206 | 0.022 | -0.551 |
| RHOG | 0.112 | 0.005 | 0.689 | NCF1C | 0.263 | 0.041 | 0.76 |
| RINL | 0.207 | 0.021 | -0.504 | NCK1 | 0.159 | 0.012 | -0.608 |
| RNASE2 | 0.293 | 0.042 | -0.624 | NCOA1 | 0.113 | 0.006 | 0.705 |
| RNF130 | 0.081 | 0.002 | 0.581 | NCOA4 | 0 | 0 | 0.787 |
| RNF145 | 0.074 | 0.002 | 0.555 | NCOA6 | 0.067 | 0.002 | 0.737 |
| RNF149 | 0.13 | 0.007 | 0.571 | NDUFA12 | 0.267 | 0.042 | -0.604 |
| RNF19A | 0.059 | 0.001 | 0.693 | NDUFA13 | 0.208 | 0.023 | -0.698 |
| RNF38 | 0.207 | 0.021 | 0.566 | NDUFB5 | 0.097 | 0.004 | -0.525 |
| RNPEP | 0.194 | 0.018 | 0.574 | NDUFS5 | 0.185 | 0.016 | -0.823 |
| ROD1 | 0.103 | 0.0045 | 0.6715 | NFAT5 | 0.028 | 0 | -0.76 |
| RPL11 | 0.165 | 0.012 | -0.641 | NFATC2IP | 0.096 | 0.004 | -0.737 |
| RPL27 | 0.16 | 0.012 | -0.694 | NFE2 | 0.192 | 0.017 | 0.686 |
| RPL4 | 0.011 | 0 | -0.67 | NKTR | 0.219 | 0.027 | 0.622 |
| RPL6 | 0.314 | 0.049 | -0.524 | NME1-NME2 | 0.272 | 0.047 | -0.566 |
| RPL7L1 | 0.075 | 0.002 | -0.58 | NOMO1 | 0.261 | 0.04 | 0.511 |
| RPS17 | 0.274 | 0.035 | -0.79 | NOTCH1 | 0.208 | 0.023 | 0.64 |
| RPS27 | 0.031 | 0 | -0.847 | NPL | 0.206 | 0.021 | 0.747 |
| RPS27L | 0.174 | 0.014 | -0.568 | NSUN5 | 0.211 | 0.024 | -0.594 |
| RPS5 | 0.169 | 0.013 | -0.552 | NT5C3 | 0.218 | 0.026 | -0.655 |
| RRAS | 0.246 | 0.029 | -0.558 | NUCB1 | 0.159 | 0.012 | 0.695 |
| RUNX2 | 0.293 | 0.042 | 0.501 | NUP93 | 0.216 | 0.025 | 0.611 |
| RXRA | 0.197 | 0.019 | 0.606 | OBFC2A | 0.001 | 0 | 0.829 |
| SCYL1 | 0.24 | 0.028 | 0.519 | OCIAD2 | 0.125 | 0.007 | -0.59 |
| SDHD | 0.207 | 0.021 | 0.506 | PARP1 | 0.002 | 0 | 0.779 |
| SEC14L1 | 0.085 | 0.003 | 0.695 | PCBP1 | 0.082 | 0.003 | 0.756 |
| SEC22C | 0.129 | 0.007 | -0.626 | PCBP2 | 0.2175 | 0.026 | 0.641 |
| SELL | 0.056 | 0.001 | 0.559 | PCMTD1 | 0.178 | 0.015 | 0.683 |
| 6-Sep | 0.034 | 0.001 | -0.683 | PCYOX1 | 0.058 | 0.001 | -0.75 |
| SERF2 | 0.043 | 0.001 | -0.722 | PDE7A | 0.058 | 0.002 | 0.681 |
| SERPINA1 | 0.06 | 0.001 | 0.508 | PDLIM7 | 0.084 | 0.003 | 0.7 |
| SF1 | 0.112 | 0.005 | -0.567 | PDPK1 | 0.172 | 0.014 | -0.559 |
| SF3B5 | 0.127 | 0.007 | -0.53 | PGAM1 | 0.22 | 0.0285 | 0.5855 |
| SFRS18 | 0.307 | 0.047 | 0.511 | PGAM4 | 0.006 | 0 | 0.76 |
| SGK | 0.075 | 0.002 | 0.504 | PGCP | 0.022 | 0 | 0.8 |
| SGK1 | 0.2505 | 0.03 | 0.6695 | PGRMC2 | 0.218 | 0.026 | 0.574 |
| SGSM2 | 0.159 | 0.012 | 0.51 | PHIP | 0.186 | 0.016 | 0.683 |
| SH2D2A | 0.13 | 0.007 | -0.51 | PLCG2 | 0.142 | 0.009 | 0.716 |
| SH3KBP1 | 0.256 | 0.031 | 0.517 | PLEKHA1 | 0.016 | 0 | -0.784 |
| SIGLEC10 | 0.143 | 0.009 | 0.665 | PLSCR1 | 0.261 | 0.041 | 0.644 |
| SIGLEC14 | 0.193 | 0.018 | 0.75 | PNPT1 | 0.268 | 0.044 | -0.656 |
| SIPA1 | 0.126 | 0.007 | 0.535 | POGK | 0.271 | 0.046 | 0.592 |
| SIRPA | 0.181 | 0.015 | 0.561 | POLE4 | 0.257 | 0.039 | -0.618 |
| SIVA | 0.144 | 0.009 | -0.503 | POLR2J4 | 0.252 | 0.038 | -0.808 |
| SKIV2L | 0.214 | 0.023 | 0.529 | POTEF | 0.063 | 0.002 | 0.518 |
| SLC11A1 | 0.195 | 0.018 | 0.733 | PP14571 | 0.066 | 0.002 | 0.734 |
| SLC40A1 | 0.293 | 0.042 | 0.552 | PPIAL4A | 0.057 | 0.002 | -0.61 |
| SLC6A6 | 0.153 | 0.01 | 0.561 | PPP1R16B | 0.272 | 0.046 | -0.613 |
| SLCO3A1 | 0.153 | 0.01 | 0.517 | PPP4R1 | 0.195 | 0.018 | 0.708 |
| SMAD4 | 0.131 | 0.007 | 0.579 | PPT1 | 0.08 | 0.003 | 0.813 |
| SMAP2 | 0.223 | 0.024 | 0.597 | PRDX1 | 0.163 | 0.012 | -0.56 |
| SNRK | 0.172 | 0.013 | 0.625 | PRKCB | 0.12 | 0.006 | 0.634 |
| SOD2 | 0.091 | 0.003 | 0.723 | PRKCD | 0.173 | 0.014 | 0.663 |
| SPAG9 | 0.017 | 0 | 0.665 | PRMT2 | 0.227 | 0.029 | -0.52 |
| SPG21 | 0.306 | 0.047 | 0.564 | PRR13 | 0.109 | 0.005 | 0.745 |
| SPI1 | 0.17 | 0.013 | 0.562 | PSAP | 0.0605 | 0.0025 | 0.7705 |
| STAG3L2 | 0.127 | 0.007 | -0.619 | PSCDBP | 0.018 | 0 | 0.835 |
| STAT3 | 0.041 | 0.001 | 0.519 | PSMB1 | 0.206 | 0.022 | -0.566 |
| STX4 | 0.033 | 0 | 0.565 | PSMB10 | 0.267 | 0.044 | 0.671 |
| TACC1 | 0.213 | 0.023 | 0.558 | PSME4 | 0 | 0 | 0.761 |
| TAGLN2 | 0.102 | 0.004 | 0.517 | PTBP1 | 0.118 | 0.006 | 0.598 |
| TALDO1 | 0.022 | 0 | 0.587 | PTPRC | 0.206 | 0.021 | -0.85 |
| TCN1 | 0.016 | 0 | 0.585 | PTPRCAP | 0.177 | 0.015 | -0.69 |
| TGFBR2 | 0 | 0 | 0.73 | PVALB | 0.208 | 0.023 | -0.613 |
| TGOLN2 | 0.204 | 0.02 | 0.547 | PYCARD | 0.097 | 0.004 | -0.604 |
| TM2D3 | 0.222 | 0.024 | 0.617 | RAB11FIP1 | 0.135 | 0.008 | 0.675 |
| TMC6 | 0.234 | 0.027 | -0.524 | RAB24 | 0.279 | 0.049 | 0.608 |
| TMED7 | 0.179 | 0.015 | 0.511 | RAB27A | 0.104 | 0.005 | -0.638 |
| TMEM66 | 0.16 | 0.011 | 0.608 | RAC2 | 0.144 | 0.01 | 0.591 |
| TNFRSF10B | 0.244 | 0.029 | 0.558 | RAF1 | 0.236 | 0.032 | 0.607 |
| TNFRSF1B | 0.044 | 0.001 | 0.709 | RALBP1 | 0.032 | 0.001 | 0.725 |
| TOMM20 | 0.12 | 0.006 | 0.523 | RB1CC1 | 0.088 | 0.004 | 0.736 |
| TOMM6 | 0.198 | 0.019 | -0.597 | RBED1 | 0.125 | 0.007 | -0.616 |
| TOMM7 | 0.094 | 0.004 | -0.699 | RBL2 | 0.007 | 0 | 0.848 |
| TPT1 | 0.26 | 0.032 | -0.819 | RBM12B | 0.03 | 0.001 | -0.785 |
| TRIM25 | 0.207 | 0.021 | -0.534 | RBM33 | 0.209 | 0.024 | 0.635 |
| TRIM38 | 0.232 | 0.026 | -0.526 | RBM47 | 0.206 | 0.021 | 0.656 |
| TRIM8 | 0.284 | 0.04 | 0.536 | RERE | 0.112 | 0.005 | 0.675 |
| TROVE2 | 0.13 | 0.008 | -0.539 | RFTN1 | 0.053 | 0.001 | 0.748 |
| TSC22D3 | 0.206 | 0.0215 | 0.587 | RHOC | 0.097 | 0.004 | -0.701 |
| TST | 0.283 | 0.039 | 0.51 | RHOG | 0.137 | 0.008 | 0.744 |
| TTC27 | 0.172 | 0.013 | 0.539 | RNASE2 | 0.066 | 0.002 | -0.815 |
| TUBA1A | 0.131 | 0.007 | 0.612 | RNASET2 | 0.261 | 0.04 | 0.643 |
| TUBB | 0.293 | 0.042 | 0.522 | RNF103 | 0.209 | 0.024 | 0.582 |
| TXK | 0.001 | 0 | 0.754 | RNF114 | 0.044 | 0.001 | 0.6 |
| TXNDC17 | 0.087 | 0.003 | -0.661 | RNF13 | 0.196 | 0.019 | 0.572 |
| TXNIP | 0.247 | 0.03 | 0.529 | RNF130 | 0.275 | 0.048 | 0.528 |
| TYMP | 0.189 | 0.017 | -0.544 | RNF145 | 0.243 | 0.034 | 0.546 |
| U2AF1 | 0.044 | 0.001 | -0.608 | RNF149 | 0.272 | 0.046 | 0.663 |
| UBAP1 | 0.283 | 0.039 | 0.57 | RNF19A | 0.249 | 0.036 | 0.678 |
| UBE2H | 0.286 | 0.04 | -0.574 | RNF34 | 0.066 | 0.002 | 0.603 |
| UBE2Z | 0.007 | 0 | -0.673 | RNF40 | 0.207 | 0.022 | 0.631 |
| UBE3C | 0.151 | 0.01 | 0.568 | ROCK2 | 0.267 | 0.043 | 0.576 |
| UBXN4 | 0.177 | 0.014 | 0.639 | ROD1 | 0.106 | 0.005 | 0.763 |
| UPF2 | 0.116 | 0.006 | 0.664 | RPL11 | 0.221 | 0.027 | -0.701 |
| UQCRHL | 0.283 | 0.038 | -0.566 | RPL13L | 0.276 | 0.048 | -0.718 |
| UQCRQ | 0.16 | 0.012 | -0.646 | RPL14 | 0.262 | 0.041 | -0.636 |
| USP48 | 0.109 | 0.005 | -0.597 | RPL17 | 0.256 | 0.0385 | -0.734 |
| VNN3 | 0.007 | 0 | 0.75 | RPL18A | 0.136 | 0.008 | -0.607 |
| VPREB3 | 0.289 | 0.041 | -0.531 | RPL23 | 0.165 | 0.013 | -0.882 |
| VPS25 | 0.136 | 0.008 | -0.591 | RPL26 | 0.164 | 0.013 | -0.91 |
| VRK3 | 0.11 | 0.005 | 0.542 | RPL27 | 0.144 | 0.009 | -0.831 |
| VTI1B | 0.251 | 0.03 | -0.563 | RPL35 | 0.206 | 0.021 | -0.696 |
| WAS | 0.183 | 0.015 | 0.649 | RPL36 | 0.172 | 0.014 | -0.627 |
| YBX1 | 0.293 | 0.042 | -0.572 | RPL4 | 0.232 | 0.03 | -0.654 |
| YWHAH | 0.136 | 0.008 | 0.611 | RPS17 | 0.206 | 0.021 | -0.884 |
| ZFP106 | 0.1825 | 0.0155 | 0.5855 | RPS26 | 0.273 | 0.046 | -0.755 |
| ZMIZ1 | 0.283 | 0.039 | 0.615 | RPS26L | 0.156 | 0.011 | -0.86 |
| ZNF160 | 0.281 | 0.037 | -0.638 | RPS27 | 0.116 | 0.006 | -0.895 |
| ZNF511 | 0.016 | 0 | -0.508 | RPS27A | 0.108 | 0.005 | -0.607 |
| ZNF586 | 0.144 | 0.009 | 0.531 | RPS27L | 0.152 | 0.011 | -0.652 |
|  |  |  |  | RPS29 | 0.267 | 0.043 | -0.754 |
|  |  |  |  | RPS3A | 0.208 | 0.023 | -0.915 |
|  |  |  |  | RPS5 | 0.127 | 0.007 | -0.727 |
|  |  |  |  | RPS6KA4 | 0.183 | 0.016 | -0.639 |
|  |  |  |  | RPS6P1 | 0.21 | 0.024 | -0.79 |
|  |  |  |  | RRAGA | 0.218 | 0.026 | -0.504 |
|  |  |  |  | RRBP1 | 0.261 | 0.041 | 0.522 |
|  |  |  |  | RTEL1 | 0.255 | 0.038 | -0.791 |
|  |  |  |  | RUNDC1 | 0.041 | 0.001 | 0.696 |
|  |  |  |  | RXRA | 0.194 | 0.017 | 0.674 |
|  |  |  |  | S100P | 0 | 0 | -0.876 |
|  |  |  |  | SAMD4B | 0.275 | 0.048 | -0.529 |
|  |  |  |  | SDF4 | 0.124 | 0.007 | 0.721 |
|  |  |  |  | SEC14L1 | 0.1695 | 0.024 | 0.731 |
|  |  |  |  | SELL | 0.01 | 0 | 0.73 |
|  |  |  |  | SELS | 0.168 | 0.013 | 0.533 |
|  |  |  |  | 6-Sep | 0.193 | 0.018 | -0.678 |
|  |  |  |  | SERF1B | 0.142 | 0.009 | 0.654 |
|  |  |  |  | SERPINA1 | 0.0535 | 0.0015 | 0.6835 |
|  |  |  |  | SF1 | 0.01 | 0 | -0.772 |
|  |  |  |  | SF3A3 | 0.279 | 0.05 | -0.531 |
|  |  |  |  | SF3B5 | 0.157 | 0.011 | -0.626 |
|  |  |  |  | SFRS2IP | 0.251 | 0.037 | 0.617 |
|  |  |  |  | SFRS5 | 0.268 | 0.043 | 0.611 |
|  |  |  |  | SFRS7 | 0.224 | 0.028 | -0.556 |
|  |  |  |  | SGK | 0.126 | 0.007 | 0.638 |
|  |  |  |  | SGK1 | 0.196 | 0.0205 | 0.7295 |
|  |  |  |  | SH2D2A | 0.208 | 0.023 | -0.516 |
|  |  |  |  | SH3GLB1 | 0.255 | 0.038 | 0.611 |
|  |  |  |  | SH3KBP1 | 0.216 | 0.025 | 0.522 |
|  |  |  |  | SHCBP1 | 0.093 | 0.004 | 0.553 |
|  |  |  |  | SIGLEC10 | 0.054 | 0.001 | 0.779 |
|  |  |  |  | SIGLEC14 | 0.146 | 0.01 | 0.865 |
|  |  |  |  | SIVA | 0.09 | 0.004 | -0.66 |
|  |  |  |  | SLA | 0.211 | 0.025 | 0.695 |
|  |  |  |  | SLC31A1 | 0.244 | 0.035 | -0.553 |
|  |  |  |  | SLC39A1 | 0.276 | 0.048 | -0.605 |
|  |  |  |  | SLC6A6 | 0.155 | 0.011 | 0.672 |
|  |  |  |  | SLC7A5 | 0.168 | 0.013 | 0.712 |
|  |  |  |  | SLCO3A1 | 0.208 | 0.023 | 0.616 |
|  |  |  |  | SMAP2 | 0.066 | 0.002 | 0.774 |
|  |  |  |  | SMNDC1 | 0.085 | 0.003 | 0.65 |
|  |  |  |  | SNCA | 0.2625 | 0.0415 | 0.801 |
|  |  |  |  | SNHG5 | 0.168 | 0.013 | -0.849 |
|  |  |  |  | SNRK | 0.0785 | 0.003 | 0.73 |
|  |  |  |  | SNRPD2 | 0.12 | 0.006 | -0.717 |
|  |  |  |  | SOD2 | 0.035 | 0.001 | 0.838 |
|  |  |  |  | SON | 0.05 | 0.001 | 0.681 |
|  |  |  |  | SP3 | 0.244 | 0.035 | 0.644 |
|  |  |  |  | SPAG9 | 0.163 | 0.013 | 0.673 |
|  |  |  |  | SPEN | 0.203 | 0.02 | 0.698 |
|  |  |  |  | SPINT1 | 0.21 | 0.024 | -0.576 |
|  |  |  |  | SRP14P1 | 0.219 | 0.026 | -0.734 |
|  |  |  |  | SS18L2 | 0.067 | 0.002 | -0.639 |
|  |  |  |  | ST6GAL1 | 0.268 | 0.043 | 0.521 |
|  |  |  |  | STAG3L2 | 0.256 | 0.039 | -0.615 |
|  |  |  |  | STAT3 | 0.169 | 0.013 | 0.583 |
|  |  |  |  | STMN3 | 0.234 | 0.031 | -0.645 |
|  |  |  |  | STX4 | 0.084 | 0.003 | 0.622 |
|  |  |  |  | SYPL1 | 0.264 | 0.041 | 0.536 |
|  |  |  |  | TACC1 | 0.168 | 0.013 | 0.626 |
|  |  |  |  | TAF12 | 0.001 | 0 | -0.765 |
|  |  |  |  | TAGLN2 | 0.137 | 0.008 | 0.613 |
|  |  |  |  | TALDO1 | 0.012 | 0 | 0.721 |
|  |  |  |  | TCN1 | 0.196 | 0.019 | 0.671 |
|  |  |  |  | TCP1 | 0.266 | 0.044 | -0.542 |
|  |  |  |  | TDRD1 | 0.266 | 0.043 | -0.788 |
|  |  |  |  | TFDP1 | 0.113 | 0.006 | 0.712 |
|  |  |  |  | TGFBR2 | 0.1735 | 0.015 | 0.6545 |
|  |  |  |  | THUMPD1 | 0.12 | 0.006 | 0.777 |
|  |  |  |  | TIPARP | 0.177 | 0.015 | 0.722 |
|  |  |  |  | TLR1 | 0.219 | 0.027 | 0.709 |
|  |  |  |  | TLR4 | 0.247 | 0.035 | 0.537 |
|  |  |  |  | TM2D3 | 0.246 | 0.036 | 0.685 |
|  |  |  |  | TMBIM1 | 0.155 | 0.011 | 0.726 |
|  |  |  |  | TMED7 | 0.066 | 0.002 | 0.67 |
|  |  |  |  | TMEM140 | 0.214 | 0.025 | 0.696 |
|  |  |  |  | TMEM158 | 0.185 | 0.016 | 0.784 |
|  |  |  |  | TMEM160 | 0.194 | 0.018 | -0.547 |
|  |  |  |  | TMEM218 | 0.004 | 0 | -0.651 |
|  |  |  |  | TMEM66 | 0.149 | 0.01 | 0.717 |
|  |  |  |  | TMEM87A | 0.078 | 0.003 | 0.682 |
|  |  |  |  | TMEM9B | 0.194 | 0.019 | -0.584 |
|  |  |  |  | TNFAIP6 | 0.268 | 0.044 | 0.77 |
|  |  |  |  | TNFRSF1A | 0.012 | 0 | 0.61 |
|  |  |  |  | TNFRSF1B | 0.207 | 0.021 | 0.696 |
|  |  |  |  | TNFSF15 | 0.207 | 0.023 | -0.863 |
|  |  |  |  | TOMM20 | 0.02 | 0 | 0.712 |
|  |  |  |  | TOMM22 | 0.01 | 0 | -0.65 |
|  |  |  |  | TOMM6 | 0.238 | 0.033 | -0.615 |
|  |  |  |  | TOMM7 | 0.112 | 0.005 | -0.826 |
|  |  |  |  | TPT1 | 0.096 | 0.004 | -0.903 |
|  |  |  |  | TRAPPC1 | 0.076 | 0.003 | -0.67 |
|  |  |  |  | TRAPPC4 | 0.24 | 0.034 | -0.589 |
|  |  |  |  | TRIM38 | 0.266 | 0.044 | -0.629 |
|  |  |  |  | TRIM58 | 0.084 | 0.003 | 0.841 |
|  |  |  |  | TRIP12 | 0.164 | 0.013 | 0.62 |
|  |  |  |  | TRMT5 | 0.239 | 0.033 | 0.668 |
|  |  |  |  | TROVE2 | 0.173 | 0.014 | -0.604 |
|  |  |  |  | TSC22D3 | 0.078 | 0.0045 | 0.6695 |
|  |  |  |  | TTC27 | 0.208 | 0.023 | 0.596 |
|  |  |  |  | TUBB | 0.206 | 0.021 | 0.659 |
|  |  |  |  | TUFM | 0.172 | 0.014 | -0.575 |
|  |  |  |  | TXK | 0.008 | 0 | 0.817 |
|  |  |  |  | TXNDC17 | 0.273 | 0.047 | -0.7 |
|  |  |  |  | U2AF1 | 0.133 | 0.008 | -0.638 |
|  |  |  |  | UBA7 | 0.142 | 0.009 | 0.507 |
|  |  |  |  | UBAP1 | 0.011 | 0 | 0.825 |
|  |  |  |  | UBC | 0.107 | 0.005 | 0.642 |
|  |  |  |  | UBE2Z | 0.206 | 0.022 | -0.627 |
|  |  |  |  | UBE3C | 0.207 | 0.022 | 0.591 |
|  |  |  |  | UCRC | 0.02 | 0 | -0.696 |
|  |  |  |  | UGP2 | 0.194 | 0.018 | -0.616 |
|  |  |  |  | UNC50 | 0.266 | 0.042 | -0.508 |
|  |  |  |  | UPF2 | 0.024 | 0 | 0.815 |
|  |  |  |  | UPP1 | 0.008 | 0 | -0.726 |
|  |  |  |  | USF2 | 0.223 | 0.0305 | -0.656 |
|  |  |  |  | VPREB3 | 0.239 | 0.033 | -0.659 |
|  |  |  |  | VPS25 | 0.166 | 0.013 | -0.663 |
|  |  |  |  | VPS26B | 0.028 | 0 | 0.66 |
|  |  |  |  | WAC | 0.268 | 0.043 | 0.651 |
|  |  |  |  | WAS | 0.275 | 0.048 | 0.59 |
|  |  |  |  | WBP2 | 0.24 | 0.034 | 0.683 |
|  |  |  |  | WDR1 | 0.2585 | 0.0405 | 0.5845 |
|  |  |  |  | WDR37 | 0.044 | 0.001 | 0.757 |
|  |  |  |  | WDR40A | 0.08 | 0.003 | 0.805 |
|  |  |  |  | WDR68 | 0.158 | 0.012 | 0.624 |
|  |  |  |  | YIPF6 | 0.194 | 0.018 | 0.639 |
|  |  |  |  | YWHAB | 0.206 | 0.021 | 0.732 |
|  |  |  |  | ZBP1 | 0.271 | 0.046 | -0.661 |
|  |  |  |  | ZBTB40 | 0.085 | 0.003 | -0.723 |
|  |  |  |  | ZC3H4 | 0.25 | 0.037 | 0.587 |
|  |  |  |  | ZC3H7A | 0.12 | 0.006 | 0.631 |
|  |  |  |  | ZC3HAV1 | 0.246 | 0.035 | 0.654 |
|  |  |  |  | ZFAND5 | 0.156 | 0.011 | 0.78 |
|  |  |  |  | ZHX2 | 0.257 | 0.04 | 0.516 |
|  |  |  |  | ZMIZ1 | 0.067 | 0.002 | 0.814 |
|  |  |  |  | ZNF217 | 0.12 | 0.007 | 0.743 |
|  |  |  |  | ZNF483 | 0.21 | 0.024 | -0.906 |
|  |  |  |  | ZNF598 | 0.261 | 0.041 | -0.535 |
|  |  |  |  | ZNF69 | 0.206 | 0.022 | -0.84 |
|  |  |  |  | ZNF91 | 0.231 | 0.03 | -0.702 |
