# Supplementary material for: Defective T Memory Cell Differentiation after Varicella Zoster Vaccination in Older Individuals
Source: PLoS Pathog. 2016 Oct 20;12(10):e1005892. doi: 10.1371/journal.ppat.1005892 (PMC5072604; doi:10.1371/journal.ppat.1005892)
Supplement: S4 Table — (DOCX) [file ppat.1005892.s004.docx]

**Supplemental Table 4: Study group examined for gene expression in activated CD4 T cells**

| **Subject ID** | **Age** | **IFNγ-specific ELISPOT (per 10^5^ PBMC)** | | | |
| --- | --- | --- | --- | --- | --- |
|  |  | **Day 0** | **Day 8** | **Day 14** | **Day 28** |
| 19 | 73 | 0.63 | 40.60 | 7.60 | 2.15 |
| 22 | 72 | 0.70 | 27.20 | 6.40 | 4.60 |
| 23 | 51 | 7.20 | 110.80 | 38.20 | 17.85 |
| 31 | 61 | 4.50 | 55.20 | 30.20 | 31.55 |
| 32 | 61 | 1.05 | 22.40 | 11.10 | 12.60 |
| 33 | 57 | 10.20 | 30.70 | 23.00 | 25.30 |
| 34 | 57 | 16.20 | 90.00 | 25.80 | 20.95 |
| 35 | 58 | 2.00 | 22.50 | 16.30 | 27.80 |
| 36 | 58 | 2.50 | 20.00 | 12.20 | 25.15 |
| 37 | 54 | 3.90 | 35.60 | 8.50 | 4.35 |
| 38 | 58 | 16.80 | 260.40 | NA | 84.80 |
| 39 | 71 | 1.10 | 9.70 | NA | 1.30 |
| 40 | 57 | 2.30 | 44.50 | 30.70 | 5.75 |
| 41 | 56 | 5.88 | 74.40 | 21.85 | 12.03 |
| 42 | 56 | 18.25 | 126.20 | 27.30 | 33.50 |
| 46 | 54 | 2.50 | 26.60 | 6.80 | 2.60 |
| 47 | 74 | 0.80 | 4.50 | 0.95 | 0.33 |
