# Supplementary material for: Defective T Memory Cell Differentiation after Varicella Zoster Vaccination in Older Individuals
Source: PLoS Pathog. 2016 Oct 20;12(10):e1005892. doi: 10.1371/journal.ppat.1005892 (PMC5072604; doi:10.1371/journal.ppat.1005892)
Supplement: S5 Table — (DOCX) [file ppat.1005892.s005.docx]

**Supplemental Table 5: Gene expression modules in activated CD4 T cells that are significantly correlated with T cell responses (p<0.01)**

| **Gene expression in activated CD4 T cells before vaccination (day0)** | | | | | |
| --- | --- | --- | --- | --- | --- |
| **Change in T cell frequencies (peak/d28)** | | | **Change in T cell frequencies (d28/d0)** | | |
| **Module** | **Rho** | **p** | **Module** | **rho** | **p** |
| mitotic cell division (M6) | -0.892 | 0.000 | TBA (M116) | -0.919 | 0.000 |
| cell cycle (III) (M103) | -0.840 | 0.000 | enriched in DNA interacting proteins (M182) | 0.885 | 0.000 |
| double positive thymocytes (M126) | -0.824 | 0.000 | axon guidance (M110) | -0.848 | 0.000 |
| B cell development/activation (M58) | -0.822 | 0.000 | mitochondrial cluster (M235) | 0.834 | 0.000 |
| cell cycle (I) (M4.1) | -0.815 | 0.001 | cytoskeleton/actin (SRF transcription targets) (M145.1) | -0.811 | 0.001 |
| myeloid cell enriched receptors and transporters (M4.3) | -0.815 | 0.001 | cytoskeleton/actin (SRF transcription targets) (M145.0) | -0.802 | 0.001 |
| BCR signaling (M54) | -0.813 | 0.001 | extracellular matrix, complement (M140) | -0.775 | 0.002 |
| enriched in monocytes (I) (M4.15) | -0.807 | 0.001 | chemokine cluster (I) (M27.0) | -0.762 | 0.003 |
| transcriptional targets of glucocorticoid receptor (M74) | -0.807 | 0.001 | regulation of localization (GO) (M63) | 0.735 | 0.005 |
| chemokine cluster (II) (M27.1) | 0.785 | 0.002 | platelet activation and degranulation (M85) | -0.724 | 0.006 |
| mitotic cell cycle - DNA replication (M4.4) | -0.774 | 0.002 | myeloid, dendritic cell activation via NFkB (II) (M43.1) | -0.722 | 0.006 |
| E2F transcription factor network (M8) | -0.772 | 0.002 | TBA (M131) | -0.720 | 0.006 |
| C-MYC transcriptional network (M4.12) | -0.768 | 0.002 | TBA (M41.2) | -0.710 | 0.007 |
| cell cycle (II) (M4.10) | -0.767 | 0.002 | TBA (M211) | 0.691 | 0.010 |
| mitotic cell cycle in stimulated CD4 T cells (M4.5) | -0.758 | 0.003 |  |  |  |
| E2F1 targets (Q4) (M10.1) | -0.749 | 0.003 |  |  |  |
| mismatch repair (I) (M22.0) | -0.748 | 0.004 |  |  |  |
| enriched in DNA interacting proteins (M182) | -0.733 | 0.005 |  |  |  |
| lymphocyte generic cluster (M60) | -0.715 | 0.007 |  |  |  |
| TBA (M41.1) | 0.705 | 0.008 |  |  |  |
| mismatch repair (II) (M22.1) | -0.692 | 0.009 |  |  |  |
|  |  |  |  |  |  |
| **Gene expression in activated CD4 T cells on day 8 after vaccination** | | | | | |
| **Change in T cell frequencies (peak/d28)** | | | **Change in T cell frequencies (d28/d0)** | | |
| **Module** | **rho** | **p** | **Module** | **rho** | **p** |
| enriched in DNA interacting proteins (M182) | -0.851 | 0.000 | translation initiation (M227) | 0.707 | 0.002 |
| transmembrane and ion transporters (II) (M224) | 0.840 | 0.000 | TBA (M197) | -0.696 | 0.002 |
| Ran mediated mitosis (M15) | -0.824 | 0.000 | TBA (M26.1) | -0.686 | 0.003 |
| golgi membrane (II) (M237) | -0.799 | 0.000 | interferon alpha response (II) (M158.1) | -0.670 | 0.004 |
| mitotic cell division (M6) | -0.795 | 0.000 | heme biosynthesis (II) (M222) | 0.669 | 0.004 |
| enriched for promoter motif NATCACGTGAY (putative SREBF1 targets) (M178) | -0.792 | 0.000 | TBA (M26.0) | -0.655 | 0.005 |
| mismatch repair (I) (M22.0) | -0.786 | 0.000 | TBA (M166) | -0.653 | 0.005 |
| mitotic cell cycle in stimulated CD4 T cells (M4.5) | -0.782 | 0.000 | golgi membrane (II) (M237) | 0.645 | 0.007 |
| TBA (M80) | -0.778 | 0.000 | enriched in DNA interacting proteins (M182) | 0.641 | 0.007 |
| cell division in stimulated CD4 T cells (M4.6) | -0.775 | 0.000 | TBA (M218) | 0.635 | 0.008 |
| mismatch repair (II) (M22.1) | -0.769 | 0.001 | TBA (M177.0) | 0.632 | 0.008 |
| TBA (M98.0) | 0.762 | 0.001 | cytoskeleton/actin (SRF transcription targets) (M145.0) | 0.630 | 0.008 |
| enriched in B cells (II) (M47.1) | -0.745 | 0.001 | TBA (M80) | 0.626 | 0.008 |
| enriched in B cells (III) (M47.2) | -0.745 | 0.001 | TBA (M233) | -0.624 | 0.009 |
| TBA (M197) | 0.743 | 0.001 | transmembrane and ion transporters (II) (M224) | -0.622 | 0.009 |
| TBA (M166) | 0.738 | 0.001 | TBA (M26.2) | -0.618 | 0.010 |
| TBA (M41.1) | 0.736 | 0.001 |  |  |  |
| plasma cells & B cells, immunoglobulins (M156.0) | -0.734 | 0.001 |  |  |  |
| enriched in B cells (V) (M47.4) | -0.733 | 0.001 |  |  |  |
| enriched in B cells (IV) (M47.3) | -0.730 | 0.001 |  |  |  |
| enriched in B cells (VI) (M69) | -0.729 | 0.001 |  |  |  |
| interferon alpha response (II) (M158.1) | 0.727 | 0.002 |  |  |  |
| cell cycle (I) (M4.1) | -0.725 | 0.002 |  |  |  |
| plasma membrane, cell junction (M162.0) | 0.724 | 0.002 |  |  |  |
| enriched in B cells (I) (M47.0) | -0.722 | 0.002 |  |  |  |
| BCR signaling (M54) | -0.722 | 0.002 |  |  |  |
| cell junction (GO) (M4.13) | -0.720 | 0.002 |  |  |  |
| TBA (M180) | -0.713 | 0.002 |  |  |  |
| enriched for SMAD2/3 signaling (M97) | -0.710 | 0.002 |  |  |  |
| cell division (M37.3) | -0.704 | 0.002 |  |  |  |
| plasma cells, immunoglobulins (M156.1) | -0.699 | 0.002 |  |  |  |
| lymphocyte generic cluster (M60) | -0.699 | 0.003 |  |  |  |
| TBA (M185) | -0.696 | 0.003 |  |  |  |
| B cell development/activation (M58) | -0.689 | 0.003 |  |  |  |
| cell division - E2F transcription network (M4.8) | -0.684 | 0.003 |  |  |  |
| cell cycle (III) (M103) | -0.682 | 0.003 |  |  |  |
| signal transduction, plasma membrane (M82) | 0.681 | 0.003 |  |  |  |
| antigen processing and presentation (M200) | -0.677 | 0.004 |  |  |  |
| enriched in plasma membrane proteins (I) (M135.0) | 0.674 | 0.004 |  |  |  |
| chaperonin mediated protein folding (I) (M204.0) | -0.673 | 0.004 |  |  |  |
| enriched in naive and memory B cells (M83) | -0.664 | 0.005 |  |  |  |
| proteasome (M226) | -0.662 | 0.005 |  |  |  |
| CD28 costimulation (M12) | -0.661 | 0.005 |  |  |  |
| transmembrane and ion transporters (I) (M142) | 0.660 | 0.005 |  |  |  |
| enriched in monocytes (I) (M4.15) | -0.660 | 0.005 |  |  |  |
| integrin cell surface interactions (II) (M1.1) | 0.659 | 0.005 |  |  |  |
| Hox cluster VI (M107) | 0.655 | 0.005 |  |  |  |
| DNA repair (M76) | -0.647 | 0.006 |  |  |  |
| TBA (M41.2) | 0.643 | 0.007 |  |  |  |
| cell division (stimulated CD4+ T cells) (M46) | -0.638 | 0.007 |  |  |  |
| phosphatidylinositol signaling system (M101) | -0.636 | 0.008 |  |  |  |
| TBA (M174) | -0.634 | 0.008 |  |  |  |
| Rho GTPase cycle (M4.14) | -0.630 | 0.008 |  |  |  |
| mitosis (TF motif CCAATNNSNNNGCG) (M169) | -0.625 | 0.009 |  |  |  |
| cytoskeleton/actin (SRF transcription targets) (M145.0) | -0.622 | 0.009 |  |  |  |
| transcriptional targets of glucocorticoid receptor (M74) | -0.621 | 0.009 |  |  |  |
| TBA (M246) | -0.621 | 0.009 |  |  |  |
| cell cycle, ATP binding (M144) | -0.620 | 0.010 |  |  |  |
|  |  |  |  |  |  |
| **Gene expression in activated CD4 T cells on day14 after vaccination** | | | | | |
| **Change in T cell frequencies (peak/d28)** | | | **Change in T cell frequencies (d28/d0)** | | |
| Module | rho | p | Module | rho | p |
| TBA (M184.1) | 0.782 | 0.000 | TBA (M177.0) | 0.860 | 0.000 |
| mitotic cell cycle in stimulated CD4 T cells (M4.5) | -0.736 | 0.001 | cell cycle (III) (M103) | 0.762 | 0.000 |
| enriched for promoter motif NATCACGTGAY (putative SREBF1 targets) (M178) | -0.729 | 0.001 | cytokines - receptor cluster (M115) | 0.751 | 0.001 |
| TBA (M184.0) | 0.720 | 0.001 | mitotic cell cycle in stimulated CD4 T cells (M4.5) | 0.733 | 0.001 |
| enriched in B cells (V) (M47.4) | -0.717 | 0.001 | CORO1A-DEF6 network (I) (M32.2) | 0.728 | 0.001 |
| DNA repair (M76) | -0.689 | 0.002 | TBA (M180) | 0.726 | 0.001 |
| mismatch repair (II) (M22.1) | -0.686 | 0.002 | mitotic cell cycle - DNA replication (M4.4) | 0.726 | 0.001 |
| cell cycle (III) (M103) | -0.678 | 0.003 | cell division in stimulated CD4 T cells (M4.6) | 0.709 | 0.001 |
| mitotic cell cycle - DNA replication (M4.4) | -0.671 | 0.004 | mitochondrial cluster (M235) | 0.708 | 0.002 |
| mitotic cell division (M6) | -0.670 | 0.004 | Ran mediated mitosis (M15) | 0.706 | 0.002 |
| regulation of antigen presentation and immune response (M5.0) | -0.668 | 0.004 | golgi membrane (II) (M237) | 0.698 | 0.002 |
| TBA (M177.0) | -0.667 | 0.004 | integrin cell surface interactions (II) (M1.1) | -0.698 | 0.002 |
| E2F transcription factor network (M8) | -0.667 | 0.004 | TBA (M170) | -0.698 | 0.002 |
| TBA (M98.0) | 0.662 | 0.005 | transmembrane and ion transporters (II) (M224) | -0.697 | 0.002 |
| cell division (stimulated CD4+ T cells) (M46) | -0.660 | 0.005 | translation initiation (M227) | 0.692 | 0.002 |
| C-MYC transcriptional network (M4.12) | -0.657 | 0.005 | TBA (M184.0) | -0.690 | 0.002 |
| cell division in stimulated CD4 T cells (M4.6) | -0.649 | 0.006 | nuclear pore complex (M106.0) | 0.674 | 0.003 |
| mismatch repair (I) (M22.0) | -0.648 | 0.006 | TBA (M125) | -0.674 | 0.003 |
| PLK1 signaling events (M4.2) | -0.637 | 0.007 | extracellular matrix (III) (M2.2) | -0.667 | 0.003 |
| enriched in B cells (III) (M47.2) | -0.631 | 0.008 | Rho GTPase cycle (M4.14) | 0.656 | 0.004 |
| cytoskeleton/actin (SRF transcription targets) (M145.0) | -0.631 | 0.008 | enriched in plasma membrane proteins (I) (M135.0) | -0.651 | 0.005 |
| cell cycle (I) (M4.1) | -0.624 | 0.008 | mismatch repair (II) (M22.1) | 0.651 | 0.005 |
| chaperonin mediated protein folding (I) (M204.0) | -0.622 | 0.009 | E2F transcription factor network (M8) | 0.650 | 0.005 |
| TBA (M197) | 0.618 | 0.009 | enriched for promoter motif NATCACGTGAY (putative SREBF1 targets) (M178) | 0.650 | 0.005 |
| TBA (M170) | 0.618 | 0.009 | receptors, cell migration (M109) | 0.650 | 0.005 |
|  |  |  | cell cycle and transcription (M4.0) | 0.648 | 0.005 |
|  |  |  | transcription elongation, RNA polymerase II (M234) | 0.647 | 0.005 |
|  |  |  | TBA (M185) | 0.641 | 0.006 |
|  |  |  | cell division (M37.3) | 0.640 | 0.006 |
|  |  |  | small GTPase mediated signal transduction (M215) | 0.639 | 0.006 |
|  |  |  | T cell signaling and costimulation (M44) | 0.637 | 0.006 |
|  |  |  | TBA (M26.0) | -0.637 | 0.006 |
|  |  |  | amino acid metabolism and transport (M154.0) | -0.636 | 0.006 |
|  |  |  | TBA (source: memory B cells) (M152.2) | -0.635 | 0.007 |
|  |  |  | DNA repair (M76) | 0.634 | 0.007 |
|  |  |  | C-MYC transcriptional network (M4.12) | 0.633 | 0.007 |
|  |  |  | cell division - E2F transcription network (M4.8) | 0.632 | 0.007 |
|  |  |  | TBA (M166) | -0.630 | 0.007 |
|  |  |  | nuclear pore, transport; mRNA splicing, processing (M143) | 0.626 | 0.008 |
|  |  |  | TBA (M184.1) | -0.625 | 0.008 |
|  |  |  | TBA (M26.2) | -0.625 | 0.008 |
|  |  |  | nuclear pore complex (mitosis) (M106.1) | 0.621 | 0.008 |
|  |  |  | cell cycle (I) (M4.1) | 0.621 | 0.009 |
|  |  |  | intracellular transport (M147) | 0.620 | 0.009 |
|  |  |  | enriched in nuclear pore complex interacting proteins (M247) | -0.617 | 0.009 |
|  |  |  | T cell surface signature (S0) | 0.616 | 0.009 |
|  |  |  | KLF12 targets network (M32.3) | 0.614 | 0.009 |
|  |  |  | TBA (M72.0) | 0.612 | 0.010 |
|  |  |  | CD28 costimulation (M12) | 0.612 | 0.010 |
|  |  |  | enriched in monocytes (I) (M4.15) | 0.608 | 0.010 |
|  |  |  | mitotic cell division (M6) | 0.607 | 0.010 |
