# Supplementary material for: Defective T Memory Cell Differentiation after Varicella Zoster Vaccination in Older Individuals
Source: PLoS Pathog. 2016 Oct 20;12(10):e1005892. doi: 10.1371/journal.ppat.1005892 (PMC5072604; doi:10.1371/journal.ppat.1005892)
Supplement: S6 Table — (DOCX) [file ppat.1005892.s006.docx]

**Supplemental Table 6: Concordance of gene expression modules in predicting VZV-specific T cell responses**

| **A.** | | | | | | |
| --- | --- | --- | --- | --- | --- | --- |
| **Correlation coefficient** | **Day8_A** | **Day8_B** | **Day14_A** | **Day14_B** | **Day0_A** | **Day0_B** |
| Day8_A | 1.00 | -0.95 | 0.96 | -0.91 | 0.84 | -0.83 |
| Day8_B | -0.95 | 1.00 | -0.90 | 0.90 | -0.71 | 0.79 |
| Day14_A | 0.96 | -0.90 | 1.00 | -0.95 | 0.83 | -0.83 |
| Day14_B | -0.91 | 0.90 | -0.95 | 1.00 | -0.74 | 0.84 |
| Day0_A | 0.84 | -0.71 | 0.83 | -0.74 | 1.00 | -0.86 |
| Day0_B | -0.83 | 0.79 | -0.83 | 0.84 | -0.86 | 1.00 |
| **B.** | | | | | | |
| **Cohen's kappa** | **Day8_A** | **Day8_B** | **Day14_A** | **Day14_B** | **Day0_A** | **Day0_B** |
| Day8_A | 1.00 | 0.40 | 0.53 | 0.36 | 0.30 | 0.01 |
| Day8_B | 0.40 | 1.00 | 0.29 | 0.30 | -0.03 | 0.02 |
| Day14_A | 0.53 | 0.29 | 1.00 | 0.51 | 0.29 | 0.02 |
| Day14_B | 0.36 | 0.30 | 0.51 | 1.00 | 0.10 | 0.08 |
| Day0_A | 0.30 | -0.03 | 0.29 | 0.10 | 1.00 | 0.23 |
| Day0_B | 0.01 | 0.02 | 0.02 | 0.08 | 0.23 | 1.00 |

Gene expression in activated T cells day 0, day 8, and day 14 was analyzed for their correlation with fold change in VZV-specific T cell frequencies (A=peak/day 28, B=day 28/day0). Table S6a shows the pairwise correlation coefficients between the rho describing the correlation of module expression with outcome. Table S6b shows the concordance of p- values significant at 0.05 level measured by Cohen’s kappa.
