# Supplementary material for: Defective T Memory Cell Differentiation after Varicella Zoster Vaccination in Older Individuals
Source: PLoS Pathog. 2016 Oct 20;12(10):e1005892. doi: 10.1371/journal.ppat.1005892 (PMC5072604; doi:10.1371/journal.ppat.1005892)
Supplement: S1 Fig — (A) CD4 or CD8 T cells were depleted of PBMCs using anti-CD4 or anti-CD8 magnetic beads and the autoMACS cell separator. For undepleted cells, PBMCs were run through the cell separator without adding magnetic beads. Purity of CD4 and CD8 T cell subpopulation was assessed by flow cytometry. (B) VZV-specific T cell frequencies were determined by IFN-γ–specific ELISpot. CD4-depleted or CD8-depleted PBMCs were compared to undepleted PBMCs using paired Wilcoxon-Manny-Whitney test. The results suggest that under these culture conditions only VZV-specific CD4 T cells were detected. (C) Over the time course of 28 days after Zostavax vaccination, frequencies of global CD4 and CD8 populations did not change. A representative example is shown. (DOCX) [file ppat.1005892.s007.docx]

**
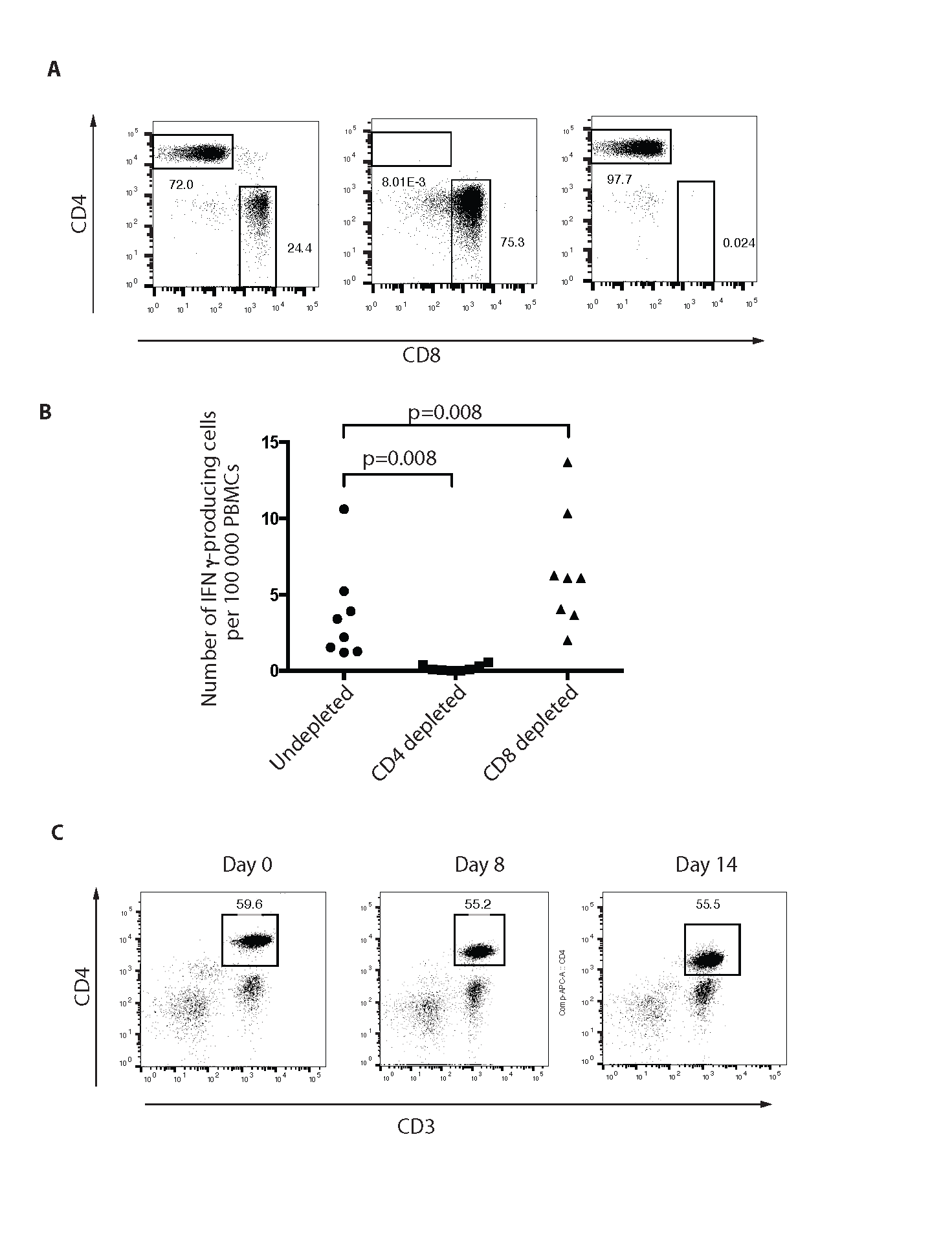
**

**Supplemental Figure 1. : Dominance of CD4 T cells in VZV-specific ELISpot assays.**  (A) CD4 or CD8 T cells were depleted of PBMCs using anti-CD4 or anti-CD8 magnetic beads and the autoMACS cell separator. For undepleted cells, PBMCs were run through the cell separator without adding magnetic beads. Purity of CD4 and CD8 T cell subpopulation was assessed by flow cytometry. (B) VZV-specific T cell frequencies were determined by IFN-γ–specific ELISpot. CD4-depleted or CD8-depleted PBMCs were compared to undepleted PBMCs using paired Wilcoxon-Manny-Whitney test. The results suggest that under these culture conditions only VZV-specific CD4 T cells were detected. (C) Over the time course of 28 days after Zostavax vaccination, frequencies of global CD4 and CD8 populations did not change. A representative example is shown.
