# Supplementary material for: Defective T Memory Cell Differentiation after Varicella Zoster Vaccination in Older Individuals
Source: PLoS Pathog. 2016 Oct 20;12(10):e1005892. doi: 10.1371/journal.ppat.1005892 (PMC5072604; doi:10.1371/journal.ppat.1005892)
Supplement: S2 Fig — Red and green nodes represent genes that positively or negatively correlated. (A) Network of genes for which the change in expression correlated with both expansion and contraction and therefore not with long-term outcome (see Venn diagram Fig 4B). (B) Network of genes informative of long-term responses. (DOCX) [file ppat.1005892.s008.docx]

**Supplemental Figure 2.** Top scoring network of monocyte-expressed genes that significantly correlated with T cell responses as shown in Figure 4 were identified using IPA software. Red and green nodes represent genes that positively or negatively correlated. (A) Network of genes for which the change in expression correlated with both expansion and contraction and therefore not with long-term outcome (see Venn diagram Figure 4B). (B) Network of genes informative of long-term responses.
