# Supplementary material for: Defective T Memory Cell Differentiation after Varicella Zoster Vaccination in Older Individuals
Source: PLoS Pathog. 2016 Oct 20;12(10):e1005892. doi: 10.1371/journal.ppat.1005892 (PMC5072604; doi:10.1371/journal.ppat.1005892)
Supplement: S3 Fig — Gene expression modules that were significantly correlated with the decline in frequencies after peak responses as well as the overall increase from day 0 to day 28 (Fig 7A), were examined for their correlation with age of study participants (S4 Table). Correlation coefficients shown as heat map exhibited a high concordance with those correlating expression levels with T cell attrition (Fig 7A). (DOCX) [file ppat.1005892.s009.docx]

**Supplemental Figure 3: Age relationship of gene expression modules that significantly correlated with T cell responses.** Gene expression modules that were significantly correlated with the decline in frequencies after peak responses as well as the overall increase from day 0 to day 28 (Figure 6F), were examined for their correlation with age of study participants (S4 Table). Correlation coefficients shown as heat map exhibited a high concordance with those correlating expression levels with T cell attrition (Figure 6F).
